# Compositional Analysis of Biomass Reference Materials: Results from an Interlaboratory Study

**DOI:** 10.1007/s12155-015-9675-1

**Published:** 2015-10-29

**Authors:** David W. Templeton, Edward J. Wolfrum, James H. Yen, Katherine E. Sharpless

**Affiliations:** 1 National Bioenergy Center, National Renewable Energy Laboratory, 15013 Denver West Parkway, MS 3512, Golden, CO 80401-3305, USA; 2 Statistical Engineering Division, National Institute of Standards and Technology, 100 Bureau Drive, Stop 8980, Gaithersburg, MD 20899-8980, USA; 3 Chemical Sciences Division, National Institute of Standards and Technology, 100 Bureau Drive, Stop 8390, Gaithersburg, MD 20899-8390, USA

**Keywords:** Biomass reference material, Compositional analysis, Sugarcane bagasse (*Saccharum* spp. hybrid) NIST RM 8491, Eastern cottonwood (*Populus deltoides*) NIST RM 8492, Monterey pine (*Pinus radiata*) NIST RM 8493, Wheat straw (*Triticum aestivum* var*. Thunderbird*) NIST RM 8494

## Abstract

Biomass compositional methods are used to compare different lignocellulosic feedstocks, to measure component balances around unit operations and to determine process yields and therefore the economic viability of biomass-to-biofuel processes. Four biomass reference materials (RMs NIST 8491–8494) were prepared and characterized, via an interlaboratory comparison exercise in the early 1990s to evaluate biomass summative compositional methods, analysts, and laboratories. Having common, uniform, and stable biomass reference materials gives the opportunity to assess compositional data compared to other analysts, to other labs, and to a known compositional value. The expiration date for the original characterization of these RMs was reached and an effort to assess their stability and recharacterize the reference values for the remaining material using more current methods of analysis was initiated. We sent samples of the four biomass RMs to 11 academic, industrial, and government laboratories, familiar with sulfuric acid compositional methods, for recharacterization of the component reference values. In this work, we have used an expanded suite of analytical methods that are more appropriate for herbaceous feedstocks, to recharacterize the RMs’ compositions. We report the median values and the expanded uncertainty values for the four RMs on a dry-mass, whole-biomass basis. The original characterization data has been recalculated using median statistics to facilitate comparisons with this data. We found improved total component closures for three out of the four RMs compared to the original characterization, and the total component closures were near 100 %, which suggests that most components were accurately measured and little double counting occurred. The major components were not statistically different in the recharacterization which suggests that the biomass materials are stable during storage and that additional components, not seen in the original characterization, were quantified here.

## Introduction

Transportation fuels derived from lignocellulosic biomass—woody material such as hardwoods, softwoods, pulp, or forestry residues and herbaceous materials such as grasses, straws, or dedicated energy crops—can reduce dependence on finite petroleum reserves, reduce anthropogenic emissions of CO_2_, and support rural economies [1, 2]. These non-food, biological materials are composed of the structural carbohydrates cellulose and hemicellulose, plus lignin and other components [3, 4]. If the structural carbohydrates found in lignocellulosic biomass can be efficiently solubilized, they can be converted via fermentation, catalysis, or other routes to fuels [5]. The resistance of biomass to degradation and solubilization is termed recalcitrance, and this is a large barrier to overcome for economical fuel production [6, 7]. Low-cost, high-volume products, such as transportation fuels, require high feedstock-to-fuel conversion yields as critical to process economics [8, 9]. For biochemical conversion pathways, a high monomer sugar yield is also a critical value [10, 11]. These summative compositional analysis methods are used to determine the effect of biomass variation on minimum ethanol selling price (MESP) [12] and to determine causes of uncertainty in MESP [13]. The determination of these conversion yields depends on high-quality analytical data for feedstock and process intermediate samples taken throughout the biofuel conversion process [14]. These sulfuric acid hydrolysis methods are useful for determining the total amount of carbohydrates available in feedstocks and process intermediate samples, but not for determining the polymer source or carbohydrate linkages within the materials. Thus, glucose released from starch, cellulose, or hemicellulose, when hydrolyzed together, is not distinguished by source but is counted in the total pool of glucan available for conversion. These biomass methods are used to compare compositions of different lignocellulosic feedstocks, to measure component balance around biorefinery unit operations and to determine conversion yields in biomass-to-biofuel processes. These data can be used to develop or improve biofuel production processes by identifying better feedstocks and conversion pathways.

A suite of analytical methods, both gravimetric and instrumental, is needed to account for all the different components (such as extractives, structural carbohydrates, lignin, protein, and ash) found in lignocellulosic biomass [15–17]. The main challenge with these biomass methods is to separately isolate and quantify each component individually without any double counting. One suite of methods, used at the National Renewable Energy Laboratory (NREL) and described elsewhere [18], is based on sulfuric acid wood lignin isolation methods and has been adapted, by NREL and others, to measure the complete composition of herbaceous lignocellulosic biomass. When adapting the analytical methods from woody to herbaceous feedstocks, new tests were needed to measure additional components that are often found in herbaceous material. Other analytical methods exist to analyze biomass for different purposes such as neutral detergent fiber/acid detergent fiber (NDF/ADF) extractions; for animal feed quality determination, different carbohydrate hydrolysis techniques using trifluoracetic acid (TFA) or HCl; for carbohydrate linkage determination, acetyl bromide or thioglycolate for lignin determinations and holocellulose methods; for wood pulping and papermaking. These different biomass methods utilize different chemical means to separate and quantify the biomass components and can give different compositional results for the same materials. They are useful for their respective industries, but these data are not interchangeable with each other and with sulfuric acid methods often used for biomass-to-biofuel processes. These sulfuric acid analytical methods are empirical, as small technique differences can affect the final result, and interrelated, as some results are used to calculate or adjust other component values. These methods can produce variable results due to differences in analysts, laboratories, and techniques [19]. With these complicated, multistep analytical methods, generating useful analytical data is difficult, though possible using proper analytical controls. The reference materials (RMs) described in this paper can provide quality control (QC) for compositional data; for instance, at NREL, one of these RMs is run with every sample batch to help demonstrate consistent compositional data.

The quality of biomass compositional data can be assessed by attaining near 100 % total component closure within a sample and by closing process component balances entering and exiting a process step. Having a common, uniform, and stable biomass reference material gives the opportunity to assess compositional data compared to a known value. It is easy to generate local QC samples to check for data variability and unexpected analytical trends but is difficult to compare such local data with outside labs. Because of the methods’ variability, reference materials (RMs) are needed for comparing compositional data among analysts, among laboratories, and over time as QC samples, as well as for assigning values to in-house QC materials.

Four lignocellulosic biomass, RMs were prepared by the International Energy Agency (IEA), National Institute of Standards and Technology (NIST), and National Renewable Energy Laboratory (NREL) in the early 1990s to aid in evaluating compositional analytical methods. These are thought to be a diverse set of biomass-to-fuel feedstocks. NIST RM 8491 sugarcane bagasse (*Saccharum* spp. hybrid), RM 8492 eastern cottonwood (*Populus deltoides*), RM 8493 Monterey pine (*Pinus radiata*), and RM 8494 wheat straw (*Triticum aestivum* var*. Thunderbird*) were originally characterized in an interlaboratory study coordinated by the IEA and NREL and reported in 1992 [20–22]. The mean (average) compositions determined in this study were used to determine the reported reference values for these RMs. The Standard Reference Materials Program at NIST serves as the distributor of these materials. Because NIST made no measurements on these four materials, reference (rather than certified) values were assigned, and the materials were categorized as Reference Materials rather than as Standard Reference Materials® [23]. The expiration date for these materials was reached in June 2010, and an effort to recharacterize the materials was initiated that year by NREL and NIST. This recharacterization was used to test for any changes in the biomass materials, using the latest compositional methods, and to revalidate the reference data reported with the RMs.

To recharacterize the four biomass RM compositions, we sent them to 11 academic, industry, and government laboratories for compositional testing. In the original 1992 characterization of the RMs, a smaller suite of analytical methods was used to determine the compositions. In this work, we have used an expanded suite of methods that are more appropriate for herbaceous feedstocks to recharacterize the RMs’ compositions and ensure that the biomass material has not degraded during storage. We used median statistics, which do not require the removal of unusual data, to determine reference compositional values among the variable data reported. Compositional data from this interlaboratory study was used by NIST to report the updated reference values for these four biomass RMs. We also have recalculated the original characterization data using median statistics (rather than mean statistics) in order to compare the two characterizations on a common statistical basis.

## Materials and Methods

### Description of Biomass RMs

Detailed descriptions of the biomass sources and sample preparations for the four biomass RMs can be found elsewhere [20]. Briefly, each of the biomass materials was hammer-milled until coarsely ground, knife-milled (through a 2-mm screen for herbaceous feedstocks and through a 1-mm screen for woody feedstocks), sieved (retaining the −20/+74 mesh fraction), homogenized in a cone blender for 45 min, irradiated with cobalt-60 (sterilized to expedite international shipment), and packaged in 10-g lots in Mylar bags. In all cases, during the knife milling operation, the cutting blades were continuously water-cooled to prevent overheating of the materials. These materials are available for purchase from NIST [24]. Samples were tested for homogeneity at two stages during the original sample preparation and packaging as described previously [20]. The bagged material was used for the original characterization testing, reported elsewhere [20–22] and distributed by NIST as RMs [24]. These RMs are stored dry and sterile in Mylar bags at room temperature, and we continue to assume that the samples are homogeneous, so samples were not randomized when they were removed from existing sales stock for recharacterization.

### Participating Labs

Biomass RM samples were sent to 11 labs (Auburn University; Audubon Sugar Institute, Louisiana State University Agricultural Center; Forest Products Laboratory; Idaho National Laboratory; Microbac Labs [Hauser Division]; Monsanto Corporation; NREL; Oregon State University; University of British Columbia; University of California, Riverside; and US Department of Agriculture, Agricultural Research Service) for compositional analysis. These labs were chosen because they regularly perform these sulfuric acid hydrolysis biomass compositional methods. Each lab was sent five packets of each of the four RMs, and they were asked to independently analyze samples from three of the packets for each of the RMs. Two analysts in two independent labs at NREL ran two sample groups, and three analysts from the same laboratory in the Riverside group provided three data sets. As many as 13 groups of compositional data (mainly triplicates) were included in the data set for each RM.

### Analytical Methods

The participating labs were asked to analyze the samples according to their normal sulfuric acid compositional methods, including their own quality control methods and component calculation algorithms. The exact details of each lab’s hydrolysis or other analytical techniques were not reported or compared for this work, although picking experienced labs that regularly run these methods was expected to reduce the variations within this data set. The methods used by NREL have been described elsewhere [18]. Some equipment-related differences were noted between the labs, and Table 1 summarizes the self-reported methods, equipment, liquid chromatography (LC) columns, and parameters used on the bagasse RM 8491 material. Each lab used the same equipment and techniques on all four of their RMs except for the acid-soluble lignin (ASL) measurement where the labs chose different wavelength and extinction coefficients. The reported differences include differences in extraction equipment and extract concentration techniques, different carbohydrate LC separation columns, and different LC detector equipment. It was though that the laboratory equipment differences would only have a minor effect on the data. All labs were not able to run all tests due to differences in capability, instrumentation, or familiarity with some methods. NIST did not analyze samples in this study, so these results again were classified as reference values rather than as certified values [23].

An expanded suite of methods, appropriate for herbaceous biomass, was used on all samples, including the woody samples, to ensure a good comparison between RMs and ensure that all biomass components were detected. Seventeen analytes and derived values were measured or calculated in this project compared with 12 in the original characterization. Samples were first sequentially extracted with water and 95 % ethanol, and the extract solutions were evaporated and weighed to quantify the soluble portions removed from the biomass. The labs used either manual Soxhlet or instrumental extraction equipment. The total extractives values (water+ethanol extractives) were used to correct any extractives-free data back to a whole (unextracted) biomass basis. Sucrose concentrations were measured from the water extract solution then back-calculated to a solids basis. All labs used some type of LC to quantitate the hydrolyzed structural carbohydrates and acetyl groups although four different column types were used. Each lab utilized their own set of LC analytical standards for quantitation and for sugar recovery standards to correct carbohydrate destruction during hydrolysis. The structural carbohydrates (glucose, xylose, arabinose, galactose, and mannose) were reported as anhydrosugars (after correcting for hydration of the monomers during hydrolysis) separately and summed together as structural sugars. The gravimetric acid insoluble residue (AIR) is reported separately from the UV ASL to account for the extreme differences in the measurement techniques and summed together as total lignin to facilitate comparisons to previous data. The AIR was corrected for ash but not for protein. As noted in Table 1, different wavelengths and extinction coefficients were used by the different labs to determine ASL. Ash was determined gravimetrically, after combustion in a furnace, on both the whole and extractives-free material in order to avoid double counting any extractable ash material in both the ash and extractives components. Protein was estimated by measuring the nitrogen in the sample and then converting these data to protein using a nitrogen-to-protein conversion factor. Different labs chose different conversion factors to report protein, which led to high protein data variability, so nitrogen values were back-calculated and are reported instead. The total component closure is calculated as the sum of the water and ethanol extractives, sucrose, extractives-free ash, structural carbohydrates, total lignin, acetyl groups, and protein. When complete sets of data were available, the total component closure (summation of all measured components) was calculated. Some total component closure results were calculated without protein or by using whole ash instead of extractives-free ash, when these differences had a minor effect on the calculated result. All data are reported on a 105 °C dry-mass, whole-biomass basis, meaning that all the results were corrected for the small amount of moisture in the samples, plus the lignin, carbohydrate, and acetyl group results were corrected for the total extractives content removed from the samples prior to hydrolysis.

### Data Reporting

A data reporting spreadsheet was developed and sent to all participants. This allowed for a common reporting format and included a questionnaire to describe the analytical methods and equipment used. The final calculated component results were reported rather than the raw measurement data. Some labs self-identified outlier data results either due to technique problems identified during the analysis or due to analytical values that the lab believed were obvious outliers. These lab-identified outlier values are not reported in the dataset, and all reported data was used to calculate median values and uncertainties (i.e., no other data points were excluded by the study organizers). After collecting all the laboratory reporting spreadsheets, we collated the data into one combined spreadsheet and confirmed the accuracy of the results with each lab individually, i.e., a laboratory was not provided with the other laboratories’ results when they were asked to confirm their own data. Analysis of variance (ANOVA) was performed using Design-Expert (Minneapolis, MN, USA) at a significance level of 0.05.

### Median Statistical Methods

Since data reported from interlaboratory comparison exercises often contain outliers, it is more appropriate to use the median value from the data set rather than the mean (average) value to calculate the reference value and expanded uncertainties [25, 26]. The choice to use the median value, as the best estimate of a component’s reference value, allows the outliers to be neglected without necessitating outlier identification and removal from the data set. The component mean value (usually the mean of triplicate determinations from each lab or individual within a lab) was calculated from the data provided. When labs analyzed for and did not detect a particular component, a value of zero was assigned, and these were included in the statistical calculations. The overall median value from as many as 13 means was chosen as the reference value for each component. Thus, the overall median (middle) result from the dataset was selected as the reference value, and any outliers would not bias the reference value based on extreme distance from the median value. Because each component reference or derived value was calculated separately, the reference component results in Table 2 are not additive. For example, the reference value for total carbohydrates is the median result of that set of values and not the summation of reference values for the individual carbohydrates.

The deviations of the lab results from the median value were used to calculate a robust estimate of the expanded uncertainty using a function of the median absolute deviation, the MADe [25, 26]. The expanded uncertainty, *U*, is calculated as *ku*_c_, where *u*_c_ incorporates the observed difference between the laboratory results, consistent with the ISO Guide and with its Supplement 1 [27, 28], and *k* is a coverage factor corresponding to approximately 95 % confidence for each analyte. Median statistics were calculated using the statistical computing language R [29], and this code was used to calculate the median statistics on the original (1992) characterization data to facilitate comparisons between the two data sets.

## Results and Discussion

### Reference Compositional Values

**Fig. 1:**
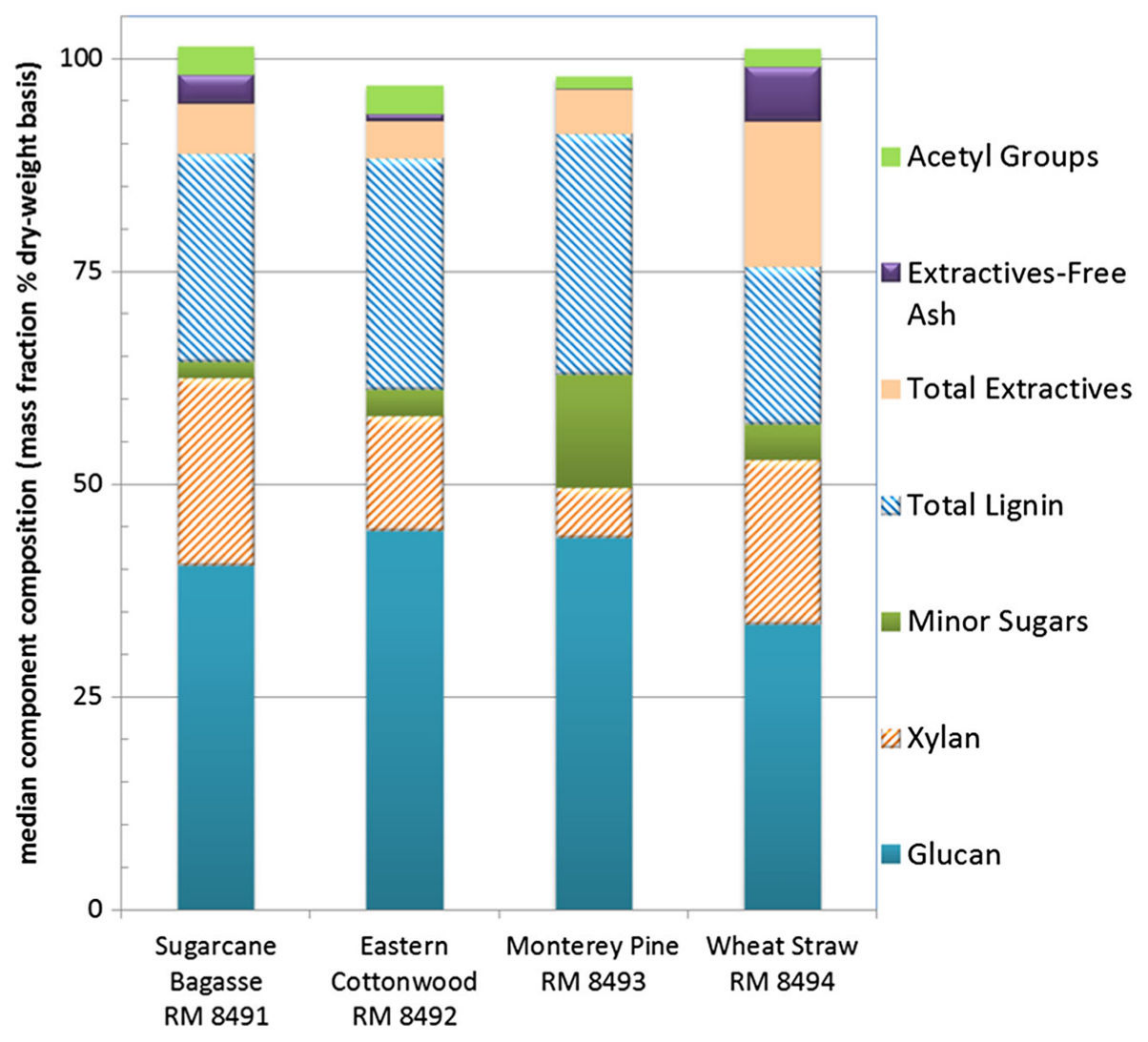
Comparison of the median, summative compositions for the four biomass RMs

Table 2 shows the median values and expanded uncertainties for the components measured in the recharacterization of the four biomass RMs. These are the reference values that are provided in the NIST Reports of Investigation supplied with each of the RMs. The position of the second, non-zero figure in the uncertainty sets the position of the last significant figure presented for the median value. As seen in Fig. 1, these biomass RMs, representing four different biomass classes, show different compositions. For instance, the softwood Monterey pine contains high amounts of mannan, often considered a minor sugar in other samples, and wheat straw contains high amounts of extractives-free ash and total extractives. Thus, the choice of which methods to include in a method suite varies depending on the specific biomass material being analyzed. In this case, one expansive suite for herbaceous feedstocks was run on all four RMs, even though negligible amounts of some components were seen. These analytical tests for negligible components could be omitted for routine testing of such biomass materials.

These four biomass RMs were originally characterized in an interlaboratory study in conjunction with the International Energy Agency (IEA), NREL, and NIST [20–22]. Twenty-three international labs participated, and the mean (average) compositional data from this previous work (presented in Table 3) was the basis for the original reference values. We have recalculated this original data using median statistics as opposed to the original mean statistics, and this data is presented in Table 4. For the sugarcane bagasse (RM 8491), an additional comparison can be made. This material was analyzed at NREL in two independent laboratories by seven analysts as part of an experiment to determine the uncertainties of the feedstock compositional methods [19].

### Total Component Closure Results

Near 100 % total component closure was seen here for all four recharacterized biomass RMs, which suggested that most feedstock components were measured and little double counting of components occurred. This gives added confidence that the individual component values, determined using many different methods, are accurate since collectively all the biomass has been measured. The recalculated median total component closures seen in the original study (88.8±9.8, 86.4±8.8, 92.0±5.3, and 95.2±8.1 % for bagasse, cottonwood, pine, and wheat straw, respectively) were significantly lower than seen here in the recharacterization study (102.4± 1.8, 99.4±1.5, 100.2±1.2, and 100.9±5.4 %, in the same order) for three out of the four RMs. The wheat straw components showed wide uncertainties, perhaps due to variability in the extractives data, which meant that the difference between the original and recharacterized total component closures was not statistically significant. Only the bagasse RM showed a total component closure greater than 100 %, which suggests that one or more of the components is biased high or some components may have been double counted. It is possible that fortuitous overcounting or undercounting of various components could sum to near 100 % or that other unknown components may not have been analyzed by this suite. Theoretically, the total component closure is bounded by 0 and 100 % although this derived value is calculated as the sum of individually analyzed components that can each be overestimated or underestimated which can lead to closure values greater than 100 %.

The improvements seen in total component closures between the original characterization and this data are partially due to measurement of additional components (larger suite of methods) run in this study. The original characterization measured fewer components and derived values (12) than this work does (17). Here we analyzed for water extractives, sucrose, extractives-free ash, structural sugars, acetyl groups, and nitrogen (for conversion to protein), components that were not measured in the original characterization. Water extractives methods in addition to 95 % ethanol extractives were run in this project because water extractives can be a large component in corn stover [30] and are expected to occur in other herbaceous feedstocks. The combined water and ethanol extractives run with the recharacterization removed significantly more extractable material here than the 95 % ethanol extraction alone did in the original study. We added methods for acetyl groups, which added 1.40 to 3.3 % to the total component closure compared to the original characterization where this component was not measured. We added a measurement for sucrose in the recharacterization suite although the only RM with significant concentrations was the wheat straw sample. In samples with high water extractives, sucrose can be a major component within the water extract and high sucrose values can interfere with the glucan measurement if sucrose is not removed in the extraction step. The original characterization included a test for glucuronic acid, which we did not repeat in this work. The spectrophotometric glucuronic acid method gave extremely variable results (±80 %) in the original characterization, and this component has not been commonly measured in lignocellulose recently. Had we seen similar values for glucuronic acid (with median values ranging from 0 to 1.2 %), the total component closure results would have increased to more than 100 %.

### Comparison to Previous RM Characterization

**Fig. 2:**
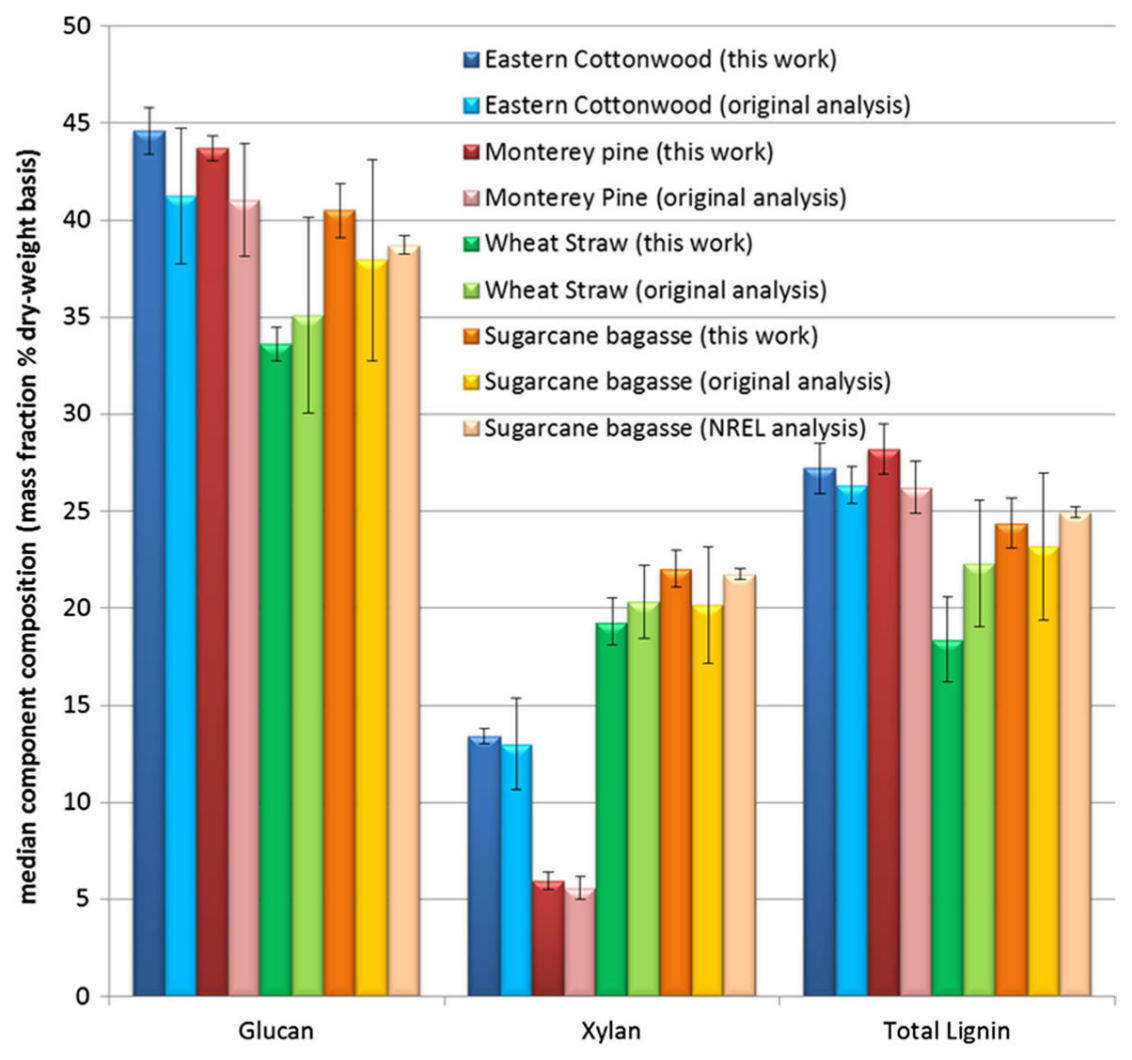
Median RM compositions of major components in this work original compared to other characterizations. *Error bars* indicate the expanded uncertainty

Figure 2 shows the comparison of the major components determined in this recharacterization and the recalculated median values from the original characterization for the four RMs. For the sugarcane bagasse RM, an additional comparison with previous NREL data is also presented. The differences between the reference values for most components are within the expanded uncertainty limits and thus are statistically unchanged between the characterizations. The only exceptions to this are the 95 % ethanol extractives and the total lignin plus the acid-insoluble residue for the wheat straw sample. Including additional component measurements helped raise the total component closures, even though the main component values were unchanged in the recharacterization. This data suggests that these reference materials are stable and unchanged even after more than 20 years of storage. These materials were extensively prepared to be dry, homogeneous, and stable, including gamma ray sterilization, and this data suggests that the RM material composition remains consistent during storage.

### Counting (or Double Counting) of Analytes

Accurate measurements of biomass components depend on careful isolation and quantification of each component. It is easy for many components to be counted more than once using these methods. Here we measured for both protein and total lignin, although some or all of the protein may precipitate into the lignin residue and be counted in the gravimetric lignin measurement. It would be useful to determine explicitly how much protein survives the lignin/carbohydrate hydrolysis to be measured in the lignin. A conservative estimation of lignin can be made by subtracting the structural protein (extractives-free protein) value from the AIR value. In this recharacterization, we used extracted ash instead of the whole ash value in the total component closure. Extracted ash is useful to avoid double counting extractable ash (the difference between whole ash and extractives-free ash) both in the whole ash and in the water extractives categories. This effect is seen in the wheat straw sample containing high extractable ash, where the total component closure value would be higher if calculated using the whole ash value. Sucrose is measured from the water extract solution, and when it is at a high concentration, it can be counted both individually and in the water extractives. The sucrose concentrations reported here were generally negligible, except for wheat straw, so this was not an issue here. This can be corrected by subtracting the sucrose amount from the water extractives value.

### Uncertainty Comparisons

The expanded uncertainties for glucan and xylan were wider in the original data than were seen here for the recharacterization, and the total lignin uncertainties are similar to the original characterization. This may be due to the greater number of laboratories involved and may also be due to the variety of carbohydrate methods used in the original characterization. The original characterization used a combination of gas chromatographic (GC) and LC carbohydrate methods for carbohydrate quantitation. The authors were surprised by the agreement between these very different carbohydrate detection systems. All the recharacterization carbohydrate data were generated using LC methods, although with different equipment and no GC data was included here. Since the original characterization, LC methods for carbohydrates have become more popular due to easier sample preparation and no need for derivatization. The wheat straw sample showed the largest expanded uncertainties among the feedstocks for most components. This may be due to the high uncertainties in water and ethanol extractives measurements plus the high ash content. Uncertainties in these values may propagate into the other components when they are corrected to a whole basis.

### Mean Biomass RM Results

The individual data points for all labs are reported as supplementary data (Tables S1, S2, S3, and S4) separated by biomass type. These tables also report the overall component means and percent relative standard deviations (%RSD) calculated based on all data points, as opposed to the overall median values reported above. These data show lower RSDs (generally <10 %) for the major components (glucan, xylan, structural sugars, total lignin, acid-insoluble residue, and total component closure) and much higher RSDs (all >20 % and some >100 % RSD) for the remaining minor components.

The two lignin components (ASL and AIR) do not show similar uncertainties. The ASL data show uncertainties of >50 % RSD while the total lignin and AIR data show uncertainties of <15 % RSD. The lab results agreed more closely for the gravimetric AIR test than for the UV–vis absorbance-based ASL measurement. Differences in wavelength and in extinction coefficients used by different labs explain part of the high ASL uncertainty. Common wavelength and extinction coefficient conditions for ASL measurement on different feedstocks need to be developed. The high uncertainty also suggests that the UV method is not specific for lignin and may be subject to interferences from other UV-absorbing compounds. Lignin is often reported as a total lignin (ASL+ AIR) value which obscures these different error sources. It is recommended that ASL and AIR values should be reported separately and not as a combined total lignin value in order to differentiate between these tests.

Different nitrogen-to-protein (N-to-P) conversion factors were used by different labs, which expanded the %RSD values for protein. To better account for the uncertainty in the underlying nitrogen method, we back-calculated and reported the nitrogen data. This highlights the need to identify a common N-to-P conversion factor for these biomass feed-stocks. Sucrose in the water extract was detected at a mass fraction of <0.10 % for three of the biomass samples and only detected at 1.35 % for wheat straw. This component has been detected at higher levels in corn stover [30]. Sucrose can be an important component in other herbaceous samples but is not normally seen in woody samples. For this RM, the sugarcane bagasse was washed nearly free of sucrose.

### Interlaboratory Comparisons

**Fig. 3:**
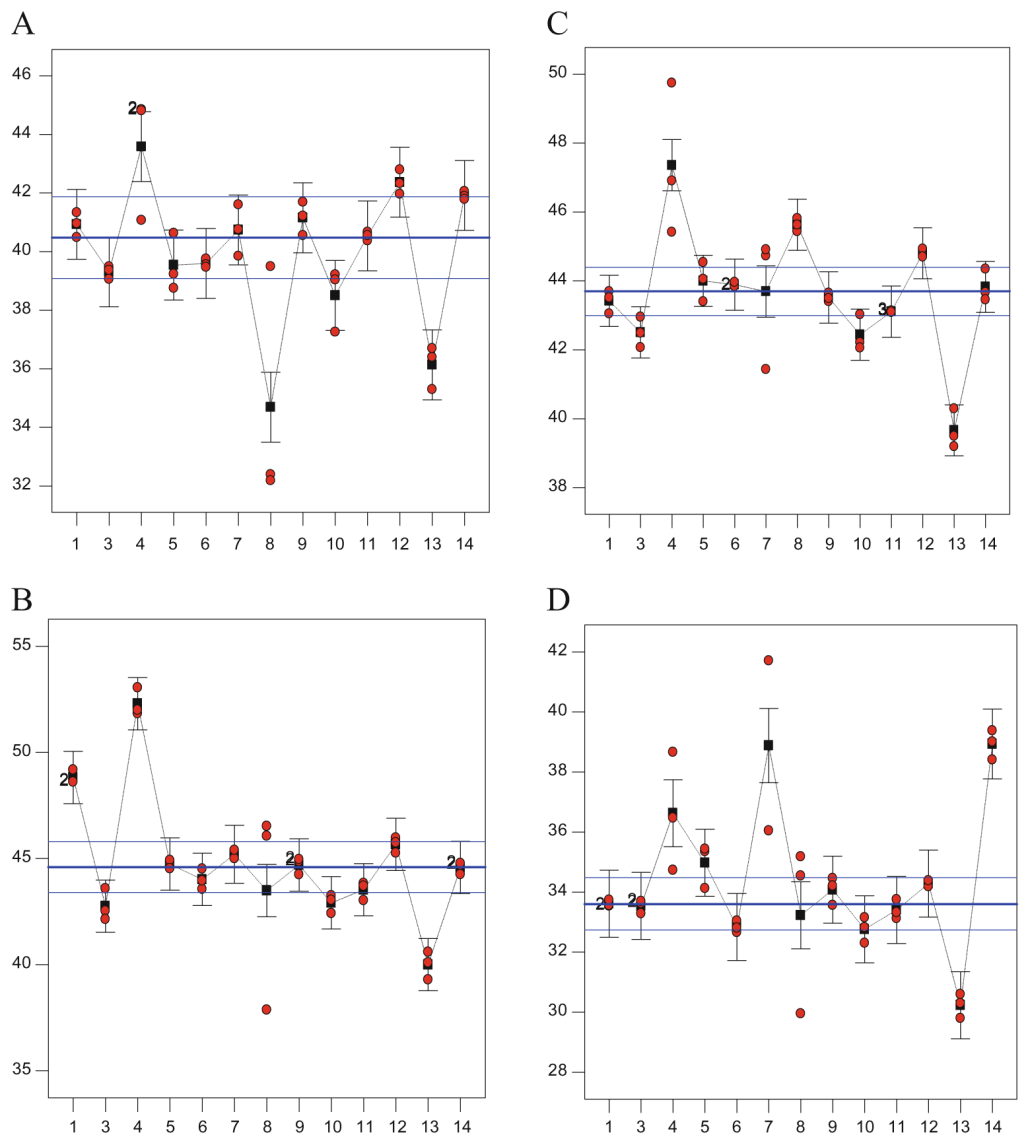
ANOVA laboratory comparison. Calculated mean glucan values are shown as *squares*, individual glucan points are *circles*, the *error bars* are least significant difference based on the entire dataset, the *horizontal thick line* depicts the median value determined from this data, and the *two thin horizontal lines* depict the expanded uncertainty range determined from this data. *Numerals* near *circles* refer to the number of overlapping points depicted. All glucan values are reported on a 105 °C dry-mass, whole-biomass basis. Panel (**a**) is sugarcane bagasse RM 8491, (**b**) is eastern cottonwood RM 8492, (**c**) is Monterey pine RM 8493, and (**d**) is wheat straw RM 8494

Figure 3 shows the individual results from each laboratory for glucan in all four RMs presented by laboratory, and the within-laboratory error bars are calculated from an ANOVA test. Most of the replicate data agreed well within a laboratory although differences between laboratories can be clearly seen. Laboratory 4 shows glucan values consistently higher and laboratory 13 shows glucan values consistently lower than the expanded uncertainty values for all four RMs. This suggests a bias in these laboratory compositional data affecting all feedstocks. Laboratory 8 shows high variability in the individual data points for the four feedstocks. Most other laboratories showed at least one feedstock glucan value outside the expanded uncertainty levels. Between four and six laboratories for each RM produced data outside the expanded uncertainty levels. Only laboratories 6, 9, and 11 had glucan values within the expanded uncertainty levels for all four RMs, while laboratories 1 (high for eastern cottonwood), 5, and 7 (both high for wheat straw) showed glucan values with uncertainty levels for three out of the four RMs. The causes of these interlaboratory differences are not known, although they could be associated by laboratory (instrumental problems, different standards), by analyst (differences in analytical technique), or by batch (autoclave run differences). This data graphically shows that it can be a common occurrence to generate extreme values using these compositional methods. With these intricate and interrelated methods, it is possible for an error in one test to affect the results in other tests. For example, incomplete drying of the removed extracts can bias the extractives result high and therefore bias the lignin and carbohydrate values low after correcting them to a whole basis. Errors in determining the sugar recovery standard values (which corrects for monomer loss during hydrolysis) can bias the carbohydrate values up or down, and incomplete hydrolysis of the biomass can bias the lignin high while simultaneously biasing the carbohydrates low. It takes a practiced eye to spot such errors in the data and attempt to determine the cause of the variability. The use of a RM during analysis can help confirm that consistent conditions have been used to generate the analytical data. The use of median statistics minimizes the effect of outlier values on the reported reference values.

### Future Work

The four biomass RMs analyzed here span different classes of feedstocks that may be converted to biofuels. Other reference materials could be developed to aid biofuel development. Possible additional reference materials include corn stover, sorghum, switchgrass, miscanthus, willow, various microalgae, macroalgae, or other aquatic plants. Potential feedstocks would need to be identified, collected in large quantities, dried, milled, and homogenized. A group of laboratories would need to be selected to analyze the feedstocks.

## Conclusions

We report here the results of an interlaboratory study to recharacterize the four NIST biomass RMs as the median component compositions and expanded uncertainties. These results were used to set the recharacterized reference values for these RMs. Total component closures calculated for these recharacterize samples were 102.4±1.8, 99.4±1.5, 100.2± 1.2, and 100.9±5.4 % (% mass fraction) for bagasse, cottonwood, pine, and wheat straw, respectively. Near 100 % component closure suggests that all the biomass components have been accurately measured. An expanded suite was used here and the extra methods measured additional components, not captured in the original characterization, which resulted in statistically higher total component closures for three out of the four RMs. The main component values were not statistically different between these two characterizations which suggests that the biomass RM materials are stable during long-term storage. Having reference materials allows for interlaboratory data comparisons of results obtained using these empirical methods. We expect that these four materials will be of continued use to the growing industry involved in identifying alternative transportation fuel processes.

## Supplementary Material

Supp1

Supp2

Supp3

Supp4

Supp5

Supp6

Supp7

Supp8

Supp9

## Figures and Tables

**Table 1:** Analytical methods, equipment, LC columns, and parameters used for characterization of sugarcane bagasse RM 8491

	Specific techniques used by the labs^a^
Extraction equipment	Soxhlet (6), automated solvent extraction (4), Soxtec (1), not reported (1)
Extract concentration technique	Rotovap (1), Turbovap (3), hotplate or oven (2), Soxtec (1), drying in crucible (1), not reported (4)
Acid-soluble lignin parameters	All labs reporting information used a wavelength of 240 nm; absorptivity of 25 L g^−1^cm^−1^ (4), absorptivity of 15 L g^−1^cm^−1^ (1), absorptivity not reported (4), no wavelength or absorptivity reported (3)
Sucrose equipment	Liquid chromatography (8), immobilized enzyme assay (3)
Sugar separation column type	Lead ion (7), amino (3), anion exchange (2), not reported (1)
LC sugar detection equipment	Refractive index (10), pulsed amperometric (2), evaporative light scattering (1)
Nitrogen method	Combustion (4), Electron Affinity (1), Kjeldahl (1), not reported (3)
Acetic acid separation column type	Hydrogen ion (8), anion (1), not reported (4)

*LC* liquid chromatography

a The value in parentheses represents the number of labs reporting the use of each method or column. Each lab used these same techniques for analysis of the other RMs, except for measurement of acid-soluble lignin where different absorptivities or wavelengths were used

**Table 2:** Median mass fraction (%)±expanded uncertainty of biomass components found in NIST biomass reference material samples on a 105 °C dry-mass, whole-biomass basis

Constituent	Sugarcane bagasse RM 8491	Eastern cottonwood RM 8492	Monterey pine RM 8493	Wheat straw RM 8494	*k*
Water extractives	4.1±1.0	2.9±1.2	3.68±0.70	15.1±2.9	2.20
95 % Ethanol extractives	1.79±0.21	1.54±0.63	1.44±0.36	2.01±0.76	2.20
Sucrose	0.10±0.03	0.045±0.046	0.030±0.035	1.35±0.68	2.20
Whole ash	3.84±0.26	0.96±0.23	0.270±0.079	9.91±0.39	2.18
Extractives-free ash	3.45±0.16	0.741±0.077	0.17±0.12	6.46±0.72	2.26
Glucan	40.5±1.4	44.6±1.2	43.7±0.66	33.61±0.87	2.18
Xylan	22.04±0.94	13.39±0.39	5.94±0.46	19.3±1.2	2.18
Arabinan	1.49±0.33	0.35±0.30	1.09±0.79	2.24±0.56	2.18
Galactan	0.28±0.33	0.55±0.53	1.89±0.31	0.62±0.47	2.20
Mannan	0.00±0.45	2.16±0.30	10.31±0.83	0.00±0.40	a
Structural sugars	65.56±0.96	61.0±1.8	62.8±3.1	56.3±1.8	2.18
Total lignin	24.4±1.3	27.2±1.3	28.2±1.3	18.4±2.2	2.18
Acid insoluble residue	20.9±1.7	24.0±1.2	25.6±1.1	15.0±1.7	2.20
Acid-soluble lignin	2.4±1.6	2.2±1.7	1.4±1.2	2.7±1.6	2.20
Acetyl groups	3.19±0.81	3.3±1.8	1.40±0.49	2.04±0.53	2.23
Nitrogen	0.21±0.10	0.17±0.11	0.160±0.062	0.54±0.23	2.31
Total component closure	102.4±1.8	99.4±1.5	100.2±1.2	100.9±5.4	b

Coverage factor (*k*) corresponding to approximately 95 % confidence for each analyte is reported for each constituent

a *k* equals 2.00 for Sugarcane bagasse and Wheat straw and 2.18 for Eastern cottonwood and Monterey pine

b *k* equals 2.26 for Sugarcane bagasse, Eastern cottonwood, and Monterey pine, and equals 2.31 for Wheat straw

**Table 3:** Mean results from original characterization of biomass RM samples

Constituent	Sugarcane bagasse RM 8491	Eastern cottonwood RM 8492	Monterey pine RM 8493	Wheat straw RM 8494
95 % Ethanol extractives	4.4	2.4	2.7	13.0
Whole ash	4.0^a^	1.0^a^	0.3^a^	10.3^a^
Glucan	38.6	42.2	41.7	32.9
Xylan	20.4	13.4	5.9	18.7
Arabinan	1.7	0.6	1.5	2.2
Galactan	0.6	0.6	2.4	0.7
Mannan	0.3	2.0	10.7	0.3
Total lignin	23.1	25.6	25.9	15.7
Glucuronic acid	1.2	3.6	2.5	1.8
Total component closure	94.3	91.4	93.6	95.6
%RSD (total)	5.5	7.3	4.5	5.4

Adapted from [21]. Reported in units of mass fraction (as %) on a 105 °C dry-mass, whole-biomass basis

a Whole ash values determined only at one lab

**Table 4:** Median mass fraction (%)±expanded uncertainty from original characterization of biomass RM samples re-calculated from data in [21]

Constituent	Sugarcane bagasse RM 8491	Eastern cottonwood RM 8492	Monterey pine RM 8493	Wheat straw RM 8494	*k*
95 % Ethanol extractives	1.45±0.36	1.15±0.24	1.30±0.12	6.35±0.48	2.07
Whole ash	4.0^a^	1.0^a^	0.3^a^	10.3^a^	n/a
Glucan	37.9±5.2	41.2±3.5	41.0±2.9	35.09±5.1	2.07
Xylan	20.2±3.0	13.0±2.3	5.59±0.59	20.3±1.9	2.07
Arabinan	1.44±0.44	0.26±0.21	1.32±0.36	2.36±0.30	2.07
Galactan	0.27±0.21	0.34±0.27	2.32±0.29	0.52±0.31	2.07
Mannan	0±0	2.05±0.52	10.5±1.0	0±0	2.07
Total lignin	23.2±3.8	26.35±0.96	26.2±1.3	22.3±3.3	2.07
Acid insoluble residue	23.15±0.44	24.45±0.64	26.55±0.48	20.7±1.1	2.07
Acid-soluble Lignin	1.05±0.72	2.00±0.88	0.40±0.32	2.05±0.60	2.07
Glucuronic acid	0.60±0.48	1.05±0.84	0±0	1.20±0.96	2.07
Total component closure	84.8±9.8	85.4±8.8	91.7±5.3	84.86±8.1	2.07

Reported in units of mass fraction (as %) on a 105 °C dry-mass, whole-biomass basis. Coverage factor (*k*) corresponding to approximately 95 % confidence for each analyte is reported for each constituent

a Whole ash values determined only at one lab

## References

[1] Ragauskas AJ, Williams CK, Davison BH, Britovsek G, Cairney J, Eckert CA, Frederick WJ, Hallett JP, Leak DJ, Liotta CL, Mielenz JR, Murphy R, Templer R, Tschaplinski T (2006). The path forward for biofuels and biomaterials. Science.

[2] Sims REH, Mabee W, Saddler JN, Taylor M (2010). An overview of second generation biofuel technologies. Bioresour Technol.

[3] Rubin EM (2008). Genomics of cellulosic biofuels. Nature.

[4] Somerville C, Bauer S, Brininstool G, Facette M, Hamann T, Milne J, Osborne E, Paredez A, Persson S, Raab T, Vorwerk S, Youngs H (2004). Toward a systems approach to understanding plant cell walls. Science.

[5] Decker SR, Sheehan J, Dayton DC, Bozell JJ, Adney WS, Hames B, Thomas SR, Bain RL, Czernik S, Zhang M, Himmel ME, Kent JA (2007). Biomass Conversion. Kent and Riegel’s handbook of industrial chemistry and biotechnology.

[6] Himmel ME, Ding S-Y, Johnson DK, Adney WS, Nimlos MR, Brady JW, Foust TD (2007). Biomass recalcitrance: engineering plants and enzymes for biofuels production. Science.

[7] Chundawat SPS, Beckham GT, Himmel ME, Dale BE (2011). Deconstruction of lignocellulosic biomass to fuels and chemicals. Annu Rev Chem Biomol Eng.

[8] Eggeman T, Elander RT (2005). Process and economic analysis of pretreatment technologies. Bioresour Technol.

[9] Tao L, Aden A, Elander RT, Pallapolu VR, Lee YY, Garlock RJ, Balan V, Dale BE, Kim Y, Mosier NS, Ladisch MR, Falls M, Holtzapple MT, Sierra R, Shi J, Ebrik MA, Redmond T, Yang B, Wyman CE, Hames B, Thomas S, Warner RE (2011). Process and technoeconomic analysis of leading pretreatment technologies for lignocellulosic ethanol production using switchgrass. Bioresour Technol.

[10] Aden A, Ruth M, Ibsen K, Jechura J, Neeves K, Sheehan J, Wallace B, Montague L, Slayton A, Lukas J (2002). Lignocellulosic Biomass to Ethanol Process Design and Economics Utilizing Co-Current Dilute Acid Prehydrolysis and Enzymatic Hydrolysis for Corn Stover.

[11] Humbird D, Davis R, Tao L, Kinchin C, Hsu D, Aden A, Schoen P, Lukas J, Olthof B, Worley M, Sexton D, Dudgeon D (2011). Process Design and Economics for Biochemical Conversion of Lignocellulosic Biomass to Ethanol: Dilute-Acid Pretreatment and Enzymatic Hydrolysis of Corn Stover. NREL.

[12] Tao L, Templeton DW, Humbird D, Aden A (2013). Effect of corn stover compositional variability on minimum ethanol selling price (MESP). Bioresour Technol.

[13] Vicari KJ, Tallam SS, Shatova T, Joo KK, Scarlata CJ, Humbird D, Wolfrum EJ, Beckham GT (2012). Uncertainty in techno-economic estimates of cellulosic ethanol production due to experimental measurement uncertainty. Biotechnol Biofuels.

[14] Dien BS, Vertes AA, Qureshi N, Blaschek HP, Yukawa H (2010). Mass balances and analytical methods for biomass pretreatment experiments. Biomass to biofuels: strategies for global industries.

[15] Hames BR, Mielenz JR (2009). Biomass compositional analysis for energy applications. Biofuels: methods and protocols. Methods in Molecular Biology.

[16] Ehrman CI, Wyman CE (1996). Methods for the chemical analysis of biomass process streams. Handbook on bioethanol: production and utilization. Applied energy technology series.

[17] Sluiter A, Sluiter J, Wolfrum EJ (2013). Methods for Biomass Compositional Analysis. Catalysis for the Conversion of Biomass and Its Derivatives.

[18] Sluiter JB, Ruiz RO, Scarlata CJ, Sluiter AD, Templeton DW (2010). Compositional analysis of lignocellulosic feedstocks. 1. Review and description of methods. J Agric Food Chem.

[19] Templeton DW, Scarlata CJ, Sluiter JB, Wolfrum EJ (2010). Compositional analysis of lignocellulosic feedstocks. 2. Method uncertainties. J Agric Food Chem.

[20] Agblevor F, Chum HL, Johnson DK, Klass DL (1993). Compositional analysis of NIST biomass standards from the IEA feedstock round robin. Energy from biomass and wastes XVI.

[21] Milne TA, Chum HL, Agblevor F, Johnson DK (1992). Standardized analytical methods. Biomass Bioenergy.

[22] Chum HL, Johnson DK, Agblevor FA, Evans RJ, Hames BR, Milne TA, Overend RP, Bridgwater AV (1992). Status of the IEA voluntary standards activity round robins on whole wood and lignins. International conference on advances in thermochemical biomass conversion.

[23] May W, Parris R, Beck C, Fassett J, Greenberg R, Guenther F, Kramer G, Wise S, Gills T, Colbert J, Gettings R, MacDonald B (2000). Definitions of Terms and Modes Used at NIST for Value-Assignment of Reference Materials for Chemical Measurements, NIST Special Publication.

[24] NIST Standard Reference Materials.

[25] Analytical Methods Committee of the RSC (1989). Robust statistics—how not to reject outliers. Part 1. Basic concepts. Analyst.

[26] Analytical Methods Committee of the RSC (1989). Robust statistics—how not to reject outliers. Part 2. Inter-laboratory trials. Analyst.

[27] ISO (1993). Guide to the Expression of Uncertainty in Measurement.

[R28] 28Evaluation of measurement data – Supplement 1 to the “Guide to the Expression of Uncertainty in Measurement” – Propagation of Distributions Using a Monte Carlo Method; Joint Committee for Guides in Metrology (BIPM, IEC, IFCC, ILAC, ISO, IUPAC, IUPAP and OIML), JCGM 101:2008, International Bureau of Weights and Measures (BIPM), Sèvres, France (2008)

[29] (2006). R: A Language and Environment for Statistical Computing.

[30] Templeton DW, Sluiter AD, Hayward TK, Hames BR, Thomas SR (2009). Assessing corn stover composition and sources of variability via NIRS. Cellulose.

