# Supplementary material for: Compositional Analysis of Biomass Reference Materials: Results from an Interlaboratory Study
Source: Bioenergy Res. Author manuscript; Available in PMC 2016 Mar 25. (PMC4807399; doi:10.1007/s12155-015-9675-1)
Supplement: Supp1 [file NIHMS740646-supplement-Supp1.doc]

**Table S1**

| Lab | Water Extr. | Ethanol Extr. | Sucrose | Glucan | Xylan | Arabinan | Galactan | Mannan | Struct. Sugars | Total Lignin | Acid-Insoluble Residue | Acid-Soluble Lignin | Protein | %N to Protein | %N | Acetyl Groups | Extr. Free Ash | Whole Ash | Total |
| --- | --- | --- | --- | --- | --- | --- | --- | --- | --- | --- | --- | --- | --- | --- | --- | --- | --- | --- | --- |
| 1 | 3.12 | 1.69 | 0.03 | 40.96 | 24.76 | 2.46 | 0 | 0 | 68.18 | 21.76 | 21.05 | 0.71 | NR | NR | NR | 2.30 | 3.54 | 3.90 | 100.59 ^a^ |
|  | 3.33 | 1.83 | 0.03 | 40.49 | 24.83 | 2.10 | 0 | 0 | 67.41 | 21.61 | 20.93 | 0.68 | NR | NR | NR | 2.23 | 3.57 | 4.04 | 99.98 ^a^ |
|  | 3.21 | 1.79 | 0.03 | 41.33 | 25.41 | 2.35 | 0 | 0 | 69.09 | 21.73 | 21.01 | 0.71 | NR | NR | NR | 2.24 | 3.55 | 3.96 | 101.62 ^a^ |
| 2 | NR | NR | NR | NR | NR | NR | NR | NR | N/A | NR | NR | NR | 2.24 | 6.25 | 0.36 | NR | NR | 3.89 | N/A |
|  | NR | NR | NR | NR | NR | NR | NR | NR | N/A | NR | NR | NR | 2.11 | 6.25 | 0.34 | NR | NR | 3.91 | N/A |
|  | NR | NR | NR | NR | NR | NR | NR | NR | N/A | NR | NR | NR | 2.21 | 6.25 | 0.35 | NR | NR | 4.12 | N/A |
| 3 | NR | NR | NR | 39.06 | 21.87 | 1.64 | 0.47 | 1.04 | 64.09 | 23.08 | 22.46 | 0.62 | NR | NR | NR | NR | NR | NR | N/A |
|  | NR | NR | NR | 39.49 | 22.14 | 1.66 | 0.49 | 1.07 | 64.85 | 23.30 | 22.66 | 0.64 | NR | NR | NR | NR | NR | NR | N/A |
|  | NR | NR | NR | 39.37 | 22.10 | 1.62 | 0.40 | 1.04 | 64.54 | 23.40 | 22.79 | 0.62 | NR | NR | NR | NR | NR | NR | N/A |
| 4 | 5.24 | 1.69 | 0 | 44.85 | 21.92 | 0.32 | 0.10 | 0.52 | 67.72 | 23.47 | 19.61 | 3.86 | 1.29 | 4.60 | 0.28 | 1.40 | 3.63 | 4.22 | 104.44 |
|  | 4.76 | 1.63 | 0 | 44.81 | 22.21 | 0.77 | 0 | 0 | 67.80 | 23.61 | 19.76 | 3.85 | 1.26 | 4.60 | 0.27 | 1.42 | 3.48 | 4.22 | 103.95 |
|  | 5.14 | 1.70 | 0 | 41.07 | 21.77 | 0.88 | 0 | 0 | 63.72 | 24.04 | 20.11 | 3.93 | 1.31 | 4.60 | 0.29 | 1.86 | 3.96 | 4.23 | 101.73 |
| 5 | 2.81 | 1.17 | 0.06 | 39.23 | 22.48 | 1.46 | 1.35 | 0.66 | 65.18 | 21.54 | 20.81 | 0.74 | 1.91 | 6.25 | 0.30 | 4.87 | NR | 3.86 | 101.34 ^b^ |
|  | 3.26 | 1.24 | 0.09 | 40.63 | 23.60 | 1.54 | 1.49 | 0.31 | 67.58 | 21.04 | 20.31 | 0.72 | 1.92 | 6.25 | 0.31 | 4.93 | NR | 4.10 | 104.06 ^b^ |
|  | 3.15 | 1.33 | 0.09 | 38.75 | 22.52 | 1.46 | 1.22 | 0.53 | 64.49 | 20.91 | 20.14 | 0.77 | 2.02 | 6.25 | 0.32 | 4.83 | NR | 4.06 | 100.79 ^b^ |
| 6 | 4.29 | 1.93 | 0.07 | 39.76 | 21.74 | 1.27 | 2.18 | 0 | 64.95 | 24.43 | 20.83 | 3.60 | 0.69 | 4.60 | 0.15 | 3.18 | 3.55 | 4.07 | 103.02 |
|  | 4.57 | 1.64 | 0.11 | 39.55 | 21.31 | 1.14 | 1.92 | 0 | 63.93 | 24.42 | 20.71 | 3.71 | 0.69 | 4.60 | 0.15 | 3.14 | 3.45 | 4.04 | 101.85 |
|  | 3.68 | 1.66 | 0.11 | 39.47 | 21.68 | 1.18 | 1.97 | 0 | 64.30 | 24.69 | 20.97 | 3.72 | 0.69 | 4.60 | 0.15 | 3.12 | 3.76 | 4.12 | 101.89 |
| 7 | 6.12 | 6.75 | 0.12 | 40.76 | 20.71 | 1.97 | NR | 1.51 | 64.94 | 26.78 | 22.59 | 4.19 | NR | NR | NR | 2.34 | NR | 3.74 | N/A |
|  | 6.81 | 7.52 | 0 | 41.60 | 20.91 | 2.16 | NR | 2.12 | 66.79 | 25.85 | 22.32 | 3.52 | NR | NR | NR | 2.73 | NR | 3.67 | N/A |
|  | 6.24 | 5.92 | 0.09 | 39.85 | 20.02 | 2.00 | NR | 2.01 | 63.88 | 25.53 | 21.50 | 4.03 | NR | NR | NR | outlier | NR | 3.36 | N/A |
| 8 | 8.42 | 2.49 | 0.12 | 39.50 | 21.68 | 1.58 | 0 | 0 | 62.75 | 24.86 | 22.77 | 2.08 | 2.18 | 6.25 | 0.35 | 5.67 | 3.42 | 3.62 | 109.79 |
|  | 8.53 | 2.76 | 0.12 | 32.39 | 18.15 | 1.30 | 0 | 0 | 51.84 | 24.59 | 22.74 | 1.85 | 1.53 | 6.25 | 0.24 | 5.49 | 3.61 | 3.48 | 98.35 |
|  | NR | NR | NR | 32.19 | 18.08 | 1.35 | 0 | 0 | 51.61 | 24.47 | 22.38 | 2.09 | 1.48 | 6.25 | 0.24 | 5.79 | 3.55 | 3.40 | N/A |
| 9 | 3.93 | 1.88 | 0.11 | 41.69 | 23.14 | 1.41 | 0.17 | 0 | 66.41 | 24.66 | 22.61 | 2.05 | NR | NR | NR | 5.24 | 3.45 | 3.60 | 105.57 ^a^ |
|  | 4.43 | 1.97 | 0.09 | 41.22 | 22.92 | 1.66 | 0.16 | 0 | 65.96 | 24.95 | 23.52 | 1.43 | NR | NR | NR | 4.66 | 3.26 | 3.62 | 105.22 ^a^ |
|  | NR | NR | NR | 40.55 | 22.56 | 1.76 | 0 | 0 | 64.88 | 25.76 | 23.86 | 1.90 | NR | NR | NR | 4.65 | 3.47 | 3.68 | N/A |
| 10 | 3.67 | 0.85 | 0.07 | 39.21 | 23.21 | 2.00 | 0 | 0 | 64.43 | 27.84 | 24.11 | 3.73 | NR | NR | NR | 5.80 | 3.50 | 4.29 | 106.09 ^a^ |
|  | 4.35 | 0.63 | 0.08 | 39.04 | 23.02 | 1.66 | 0 | 0 | 63.71 | 26.65 | 22.82 | 3.83 | NR | NR | NR | 0.10 | 3.51 | 4.30 | 98.95 ^a^ |
|  | NR | NR | NR | 37.26 | 22.54 | 1.60 | 0 | 0 | 61.39 | 22.64 | 22.10 | 0.54 | NR | NR | NR | 5.28 | 3.49 | 4.19 | N/A |
| 11 | 3.73 | 1.73 | 0.38 | 40.37 | 22.55 | 0.81 | 1.70 | 0 | 65.43 | 24.50 | 20.79 | 3.71 | 0.74 | 4.60 | 0.16 | 3.20 | 2.86 | 4.28 | 102.17 |
|  | 3.66 | 1.55 | 0.34 | 40.67 | 22.59 | 0.91 | 1.80 | 0 | 65.97 | 24.32 | 20.70 | 3.62 | 0.69 | 4.60 | 0.15 | 3.21 | 3.02 | 3.65 | 102.41 |
|  | 3.67 | 1.67 | 0.43 | 40.56 | 22.40 | 0.84 | 1.76 | 0 | 65.55 | 24.29 | 20.59 | 3.70 | 0.64 | 4.60 | 0.14 | 3.15 | 2.92 | 3.59 | 101.90 |
| 12 | 2.76 | 2.06 | 1.24 | 42.80 | 21.59 | 1.12 | 0.44 | 0.46 | 66.42 | 19.30 | 18.19 | 1.11 | 1.28 | 5.80 | 0.22 | NR | 3.29 | 3.13 | N/A |
|  | 2.54 | 1.97 | 1.14 | 42.33 | 21.15 | 1.18 | 0.45 | 0.47 | 65.58 | 20.91 | 19.85 | 1.05 | 1.22 | 5.80 | 0.21 | NR | 3.29 | 3.14 | N/A |
|  | 2.25 | 2.31 | 1.26 | 41.96 | 20.68 | 1.16 | 0.44 | 0.44 | 64.68 | 19.16 | 18.04 | 1.12 | 1.16 | 5.80 | 0.20 | NR | 3.33 | 3.17 | N/A |
| 13 | 6.10 | 2.20 | 0.10 | 35.30 | 19.20 | 1.40 | 0.80 | 0.30 | 57.00 | 21.40 | 18.70 | 2.70 | 0.80 | 6.25 | 0.13 | 2.70 | 3.40 | 3.80 | 93.60 |
|  | 5.80 | 2.10 | 0.10 | 36.40 | 19.70 | 1.40 | 0.80 | 0.20 | 58.50 | 22.10 | 19.30 | 2.80 | 0.80 | 6.25 | 0.13 | 2.80 | 3.20 | 3.90 | 95.30 |
|  | 3.00 | 1.70 | 0.10 | 36.70 | 20.20 | 1.40 | 0.90 | 0.30 | 59.50 | 22.10 | 19.10 | 3.00 | 0.80 | 6.25 | 0.13 | 3.10 | 3.60 | 3.80 | 93.80 |
| 14 | 3.89 | 1.85 | 1.18 | 42.06 | 22.31 | 1.86 | 0.00 | 0.00 | 66.23 | 25.63 | NR | NR | 0.55 | 4.60 | 0.12 | 3.33 | 3.22 | 3.65 | 104.70 |
|  | 3.78 | 1.78 | 0.58 | 41.89 | 22.35 | 1.78 | 0.00 | 0.00 | 66.02 | 25.58 | NR | NR | 0.60 | 4.60 | 0.13 | 3.38 | 3.08 | 3.47 | 104.22 |
|  | 3.91 | 1.80 | 0.45 | 41.79 | 22.20 | 1.80 | 0.00 | 0.00 | 65.79 | 25.43 | NR | NR | 0.60 | 4.60 | 0.13 | 3.38 | 3.26 | 3.70 | 104.17 |
| mean | 4.37 | 2.21 | 0.26 | 39.92 | 21.90 | 1.49 | 0.58 | 0.33 | 64.18 | 23.65 | 21.19 | 2.30 | 1.24 | N/A | 0.22 | 3.48 | 3.41 | 3.82 | 101.91 |
| stdev | 1.56 | 1.52 | 0.38 | 2.65 | 1.57 | 0.46 | 0.72 | 0.56 | 3.83 | 2.04 | 1.57 | 1.36 | 0.59 | N/A | 0.08 | 1.45 | 0.24 | 0.33 | 3.65 |
| %RSD | 36 | 69 | 144 | 6.6 | 7.2 | 31 | 123 | 167 | 6.0 | 8.6 | 7.4 | 59 | 47 | N/A | 38 | 42 | 7.0 | 8.6 | 3.6 |

NR – not run

N/A – not applicable

outlier – not reported, identified by laboratory as an unusual value

^a^ Total value calculated without protein

^b^ Total value calculated using whole ash instead of extractives free ash
