# Supplementary material for: Compositional Analysis of Biomass Reference Materials: Results from an Interlaboratory Study
Source: Bioenergy Res. Author manuscript; Available in PMC 2016 Mar 25. (PMC4807399; doi:10.1007/s12155-015-9675-1)
Supplement: Supp2 [file NIHMS740646-supplement-Supp2.doc]

**Table S2**

| Lab | Water Extr. | Ethanol Extr. | Sucrose | Glucan | Xylan | Arabinan | Galactan | Mannan | Struct. Sugars | Total Lignin | Acid-Insoluble Residue | Acid-Soluble Lignin | Protein | %N to Protein | %N | Acetyl Groups | Extr. Free Ash | Whole Ash | Total |
| --- | --- | --- | --- | --- | --- | --- | --- | --- | --- | --- | --- | --- | --- | --- | --- | --- | --- | --- | --- |
| 1 | 0.32 | 3.78 | 0.01 | 48.65 | 14.42 | 1.71 | 1.87 | 2.12 | 68.77 | 25.67 | 24.92 | 0.75 | NR | NR | NR | 1.89 | 0.70 | 0.98 | 101.13 ^a^ |
|  | 0.31 | 3.62 | 0.01 | 49.19 | 14.25 | 1.11 | 1.71 | 1.97 | 68.23 | 25.87 | 25.02 | 0.86 | NR | NR | NR | 1.99 | 0.73 | 0.94 | 100.75 ^a^ |
|  | 0.33 | 3.63 | 0.01 | 48.62 | 14.22 | 1.11 | 1.70 | 1.96 | 67.61 | 25.78 | 25.06 | 0.72 | NR | NR | NR | 1.91 | 0.85 | 0.97 | 100.12 ^a^ |
| 2 | NR | NR | NR | NR | NR | NR | NR | NR | N/A | NR | NR | NR | 1.58 | 6.25 | 0.25 | NR | NR | 1.01 | N/A |
|  | NR | NR | NR | NR | NR | NR | NR | NR | N/A | NR | NR | NR | 1.52 | 6.25 | 0.24 | NR | NR | 1.14 | N/A |
|  | NR | NR | NR | NR | NR | NR | NR | NR | N/A | NR | NR | NR | 1.52 | 6.25 | 0.24 | NR | NR | 1.00 | N/A |
| 3 | NR | NR | NR | 42.54 | 13.42 | 0.40 | 0.47 | 2.44 | 59.27 | 25.99 | 25.43 | 0.56 | NR | NR | NR | NR | NR | NR | N/A |
|  | NR | NR | NR | 42.14 | 13.32 | 0.36 | 0.53 | 2.20 | 58.55 | 25.76 | 25.20 | 0.57 | NR | NR | NR | NR | NR | NR | N/A |
|  | NR | NR | NR | 43.60 | 13.51 | 0.29 | 0.52 | 2.49 | 60.41 | 25.81 | 25.28 | 0.53 | NR | NR | NR | NR | NR | NR | N/A |
| 4 | 1.49 | 1.27 | 0 | 53.06 | 13.94 | 1.86 | 0.26 | 0.00 | 69.12 | 27.68 | 23.29 | 4.39 | 1.03 | 4.60 | 0.22 | 1.63 | 0.36 | 0.45 | 102.58 |
|  | 2.66 | 1.24 | 0 | 51.84 | 13.84 | 0 | 0.25 | 1.27 | 67.20 | 26.99 | 22.84 | 4.15 | 0.88 | 4.60 | 0.19 | 1.53 | 0.38 | 0.38 | 100.88 |
|  | 2.85 | 1.14 | 0 | 52.00 | 13.88 | 0 | 0.20 | 1.13 | 67.20 | 27.05 | 22.76 | 4.29 | 0.92 | 4.60 | 0.20 | 1.63 | 0.41 | 0.41 | 101.20 |
| 5 | 1.77 | 0.85 | 0 | 44.76 | 16.23 | 0.37 | 1.66 | 3.72 | 66.73 | 23.66 | 22.86 | 0.80 | 1.08 | 6.25 | 0.17 | 4.97 | NR | 1.22 | 100.28 ^b^ |
|  | 1.74 | 1.19 | 0 | 44.92 | 15.70 | 0.38 | 1.58 | 4.31 | 66.88 | 23.48 | 22.70 | 0.79 | 1.32 | 6.25 | 0.21 | 5.01 | NR | 1.09 | 100.72 ^b^ |
|  | 1.79 | 0.79 | 0 | 44.53 | 15.44 | 0.34 | 1.63 | 3.89 | 65.83 | 23.74 | 22.94 | 0.81 | 1.34 | 6.25 | 0.21 | 4.87 | NR | 1.25 | 99.62 ^b^ |
| 6 | 2.73 | 0.82 | 0.03 | 44.52 | 13.30 | 1.15 | 0.82 | 2.21 | 62.01 | 28.33 | 23.97 | 4.37 | 0.37 | 4.60 | 0.08 | 3.15 | 0.74 | 1.21 | 98.15 |
|  | 3.46 | 1.56 | 0.07 | 43.57 | 13.19 | 1.19 | 0.81 | 2.30 | 61.05 | 27.86 | 23.68 | 4.18 | 0.37 | 4.60 | 0.08 | 3.17 | 0.73 | 1.18 | 98.20 |
|  | 2.89 | 1.30 | 0.06 | 43.96 | 13.67 | 1.12 | 0.75 | 2.40 | 61.91 | 28.24 | 23.88 | 4.36 | 0.32 | 4.60 | 0.07 | 3.18 | 0.71 | 1.26 | 98.55 |
| 7 | 5.03 | 3.35 | 0.14 | 45.01 | 13.08 | 0 | NR | 4.04 | 62.13 | 28.68 | 24.45 | 4.23 | NR | NR | NR | 1.57 | NR | 0.54 | N/A |
|  | 6.81 | 4.92 | 0.12 | 45.40 | 13.07 | 0 | NR | 4.75 | 63.22 | 29.35 | 25.55 | 3.80 | NR | NR | NR | outlier | NR | 0.64 | N/A |
|  | 4.98 | 4.78 | 0.11 | outlier | outlier | 0 | NR | outlier | N/A | N/A | outlier | 3.36 | NR | NR | NR | 1.57 | NR | 0.59 | N/A |
| 8 | 4.95 | 4.18 | 0.04 | 37.87 | 11.89 | 0 | 0 | 1.01 | 50.77 | 24.65 | 22.55 | 2.10 | 2.20 | 6.25 | 0.35 | 6.54 | 0.63 | 1.19 | 93.92 |
|  | 4.88 | 4.61 | 0.06 | 46.07 | 13.74 | 0 | 0 | 1.47 | 61.28 | 26.58 | 24.37 | 2.21 | 1.90 | 6.25 | 0.30 | 6.13 | 0.98 | 1.05 | 106.37 |
|  | NR | NR | NR | 46.53 | 13.79 | 0 | 0 | 1.52 | 61.84 | 25.98 | 23.71 | 2.27 | 1.77 | 6.25 | 0.28 | 6.24 | 0.66 | 0.40 | N/A |
| 9 | 4.05 | 0.61 | 0.10 | 44.87 | 12.86 | 0 | 0.22 | 2.28 | 60.23 | 29.33 | 26.98 | 2.35 | NR | NR | NR | 5.77 | 0.67 | 0.86 | 100.66 ^a^ |
|  | 3.88 | 0.62 | 0.10 | 44.97 | 13.46 | 0 | 0.22 | 2.26 | 60.91 | 28.38 | 26.09 | 2.29 | NR | NR | NR | 5.69 | 0.80 | 0.86 | 100.27 ^a^ |
|  | NR | NR | NR | 44.25 | 12.65 | 0 | 0.19 | 2.15 | 59.24 | 27.54 | 25.27 | 2.27 | NR | NR | NR | 5.65 | 0.80 | 0.91 | N/A |
| 10 | 4.94 | 2.01 | 0.04 | 43.26 | 13.69 | 0.09 | 0 | 1.88 | 58.92 | 27.87 | 26.88 | 0.99 | NR | NR | NR | 4.61 | 0.51 | 1.27 | 98.86 ^a^ |
|  | 3.32 | 3.88 | 0.03 | 43.05 | 13.70 | 0.09 | 0 | 1.84 | 58.68 | 27.37 | 25.58 | 1.78 | NR | NR | NR | 4.62 | 0.52 | 1.30 | 98.38 ^a^ |
|  | NR | NR | NR | 42.42 | 13.44 | 0.08 | 0 | 1.95 | 57.90 | 28.27 | 24.99 | 3.28 | NR | NR | NR | 4.77 | 0.51 | 1.25 | N/A |
| 11 | 2.60 | 1.80 | 0.15 | 43.84 | 13.18 | 1.05 | 1.18 | 1.59 | 60.85 | 27.51 | 23.16 | 4.35 | 0.32 | 4.60 | 0.07 | 3.27 | 0.15 | 0.78 | 96.51 |
|  | 3.01 | 2.46 | 0.13 | 43.71 | 13.07 | 1.02 | 1.21 | 1.38 | 60.39 | 27.39 | 23.15 | 4.25 | 0.32 | 4.60 | 0.07 | 3.22 | 0.30 | 0.67 | 97.09 |
|  | 2.54 | 1.77 | 0.23 | 43.04 | 12.87 | 1.03 | 1.53 | 0.73 | 59.19 | 27.27 | 23.05 | 4.21 | 0.32 | 4.60 | 0.07 | 3.28 | 0.20 | 0.74 | 94.57 |
| 12 | 1.64 | 1.41 | 0.04 | 45.98 | 12.40 | 0.27 | 0.58 | 2.25 | 61.48 | 26.08 | 24.23 | 1.84 | 1.16 | 5.80 | 0.20 | NR | 0.85 | 0.82 | N/A |
|  | 1.94 | 1.38 | 0.06 | 45.27 | 12.12 | 0.27 | 0.57 | 2.22 | 60.45 | 26.32 | 24.39 | 1.93 | 0.99 | 5.80 | 0.17 | NR | 0.85 | 0.82 | N/A |
|  | 2.2 | 1.53 | 0.02 | 45.75 | 12.22 | 0.30 | 0.61 | 2.28 | 61.17 | 26.09 | 24.10 | 1.99 | 0.81 | 5.80 | 0.14 | NR | 0.79 | 0.76 | N/A |
| 13 | 3.10 | 1.80 | 0 | 40.10 | 12.10 | 0.40 | 0.90 | 1.90 | 55.40 | 26.30 | 22.50 | 3.80 | 0.50 | 6.25 | 0.08 | 3.00 | 0.80 | 1.20 | 90.90 |
|  | 4.70 | 1.60 | 0 | 39.30 | 11.90 | 0.30 | 0.90 | 1.80 | 54.20 | 25.60 | 21.90 | 3.70 | 0.60 | 6.25 | 0.10 | 2.80 | 0.70 | 1.10 | 90.20 |
|  | 2.20 | 1.40 | 0 | 40.60 | 12.30 | 0.30 | 0.90 | 1.90 | 56.00 | 26.50 | 22.90 | 3.60 | 0.60 | 6.25 | 0.10 | 3.00 | 0.80 | 1.20 | 90.50 |
| 14 | 2.64 | 1.47 | 0.3 | 44.26 | 13.21 | 0.55 | 0.00 | 2.17 | 60.19 | 28.86 | NR | NR | 0.37 | 4.60 | 0.08 | 3.31 | 0.40 | 1.04 | 97.24 |
|  | 2.64 | 1.45 | 0.25 | 44.70 | 13.32 | 0.55 | 0.00 | 2.17 | 60.74 | 29.06 | NR | NR | 0.32 | 4.60 | 0.07 | 3.42 | 0.62 | 1.03 | 98.25 |
|  | 2.65 | 1.51 | 0.45 | 44.78 | 14.64 | 0.58 | 0.00 | 2.15 | 62.15 | 28.55 | NR | NR | 0.32 | 4.60 | 0.07 | 3.38 | 0.36 | 1.06 | 98.92 |
| mean | 2.94 | 2.11 | 0.08 | 44.81 | 13.45 | 0.47 | 0.66 | 2.16 | 61.53 | 26.88 | 24.16 | 2.58 | 0.92 | N/A | 0.16 | 3.65 | 0.62 | 0.94 | 98.33 |
| stdev | 1.50 | 1.31 | 0.10 | 3.19 | 0.98 | 0.51 | 0.61 | 0.94 | 4.14 | 1.54 | 1.29 | 1.44 | 0.57 | N/A | 0.09 | 1.56 | 0.21 | 0.27 | 3.71 |
| %RSD | 51 | 62 | 128 | 7.1 | 7.3 | 108 | 93 | 44 | 6.7 | 5.7 | 5.4 | 56 | 62 | N/A | 54 | 43 | 35 | 29 | 3.8 |

NR – not run

N/A – not applicable

outlier – not reported, identified by laboratory as an unusual value

^a^ Total value calculated without protein

^b^ Total value calculated using whole ash instead of extractives free ash
