# Supplementary material for: Compositional Analysis of Biomass Reference Materials: Results from an Interlaboratory Study
Source: Bioenergy Res. Author manuscript; Available in PMC 2016 Mar 25. (PMC4807399; doi:10.1007/s12155-015-9675-1)
Supplement: Supp3 [file NIHMS740646-supplement-Supp3.doc]

**Table S3**

| Lab | Water Extr. | Ethanol Extr. | Sucrose | Glucan | Xylan | Arabinan | Galactan | Mannan | Struct. Sugars | Total Lignin | Acid-Insoluble Residue | Acid-Soluble Lignin | Protein | %N to Protein | %N | Acetyl Groups | Extr. Free Ash | Whole Ash | Total |
| --- | --- | --- | --- | --- | --- | --- | --- | --- | --- | --- | --- | --- | --- | --- | --- | --- | --- | --- | --- |
| 1 | 3.97 | 1.04 | 0.01 | 43.70 | 7.94 | 0 | 0 | 11.52 | 63.16 | 31.08 | 30.64 | 0.44 | NR | NR | NR | 0.78 | 0.13 | 0.28 | 100.16 ^a^ |
|  | 3.99 | 1.06 | 0.01 | 43.52 | 8.13 | 0 | 0 | 11.56 | 63.21 | 30.81 | 30.37 | 0.45 | NR | NR | NR | 0.75 | 0.14 | 0.30 | 99.96 ^a^ |
|  | 4.03 | 1.18 | 0.01 | 43.05 | 7.85 | 0 | 0 | 11.25 | 62.15 | 30.84 | 30.35 | 0.48 | NR | NR | NR | 0.77 | 0.13 | 0.29 | 99.10 ^a^ |
| 2 | NR | NR | NR | NR | NR | NR | NR | NR | N/A | NR | NR | NR | 1.37 | 6.25 | 0.22 | NR | NR | 0.29 | N/A |
|  | NR | NR | NR | NR | NR | NR | NR | NR | N/A | NR | NR | NR | 1.12 | 6.25 | 0.18 | NR | NR | 0.28 | N/A |
|  | NR | NR | NR | NR | NR | NR | NR | NR | N/A | NR | NR | NR | 1.19 | 6.25 | 0.19 | NR | NR | 0.28 | N/A |
| 3 | NR | NR | NR | 42.96 | 6.39 | 1.58 | 2.36 | 10.99 | 64.28 | 26.19 | 25.69 | 0.49 | NR | NR | NR | NR | NR | NR | N/A |
|  | NR | NR | NR | 42.49 | 5.77 | 1.54 | 2.29 | 10.03 | 62.13 | 27.78 | 27.28 | 0.51 | NR | NR | NR | NR | NR | NR | N/A |
|  | NR | NR | NR | 42.07 | 5.76 | 1.51 | 2.17 | 10.19 | 61.70 | 27.83 | 27.33 | 0.50 | NR | NR | NR | NR | NR | NR | N/A |
| 4 | 3.12 | 1.29 | 0 | 45.42 | 5.95 | 0.13 | 2.86 | 12.26 | 66.62 | 29.12 | 25.22 | 3.9 | 0.71 | 4.60 | 0.15 | 1.20 | 0.25 | 0.35 | 102.29 |
|  | 3.06 | 1.39 | 0 | 46.91 | 5.15 | 0.52 | 1.59 | 9.41 | 63.58 | 28.77 | 24.99 | 3.78 | 0.89 | 4.60 | 0.19 | 0.95 | 0.29 | 0.33 | 98.94 |
|  | 3.22 | 1.42 | 0 | 49.75 | 5.49 | 0.45 | 1.27 | 9.25 | 66.21 | 28.40 | 24.78 | 3.62 | 0.79 | 4.60 | 0.17 | 0.95 | 0.29 | 0.37 | 101.28 |
| 5 | 2.27 | 0.71 | 0 | 43.40 | 7.26 | 1.41 | 3.30 | 13.09 | 68.47 | 25.62 | 25.14 | 0.48 | 0.94 | 6.25 | 0.15 | 2.09 | NR | 0.27 | 100.36 ^b^ |
|  | 2.61 | 0.93 | 0 | 44.06 | 7.38 | 1.40 | 3.21 | 14.10 | 70.14 | 25.11 | 24.77 | 0.34 | 1.05 | 6.25 | 0.17 | 2.01 | NR | 0.25 | 102.10 ^b^ |
|  | 2.03 | 0.58 | 0 | 44.54 | 7.31 | 1.48 | 3.19 | 13.40 | 69.93 | 25.16 | 24.83 | 0.33 | 0.97 | 6.25 | 0.16 | 2.00 | NR | 0.29 | 100.96 ^b^ |
| 6 | 3.64 | 1.31 | 0.12 | 43.87 | 6.55 | 2.52 | 1.83 | 10.78 | 65.54 | 28.91 | 25.47 | 3.44 | 0.20 | 4.60 | 0.04 | 1.34 | 0.19 | 0.61 | 101.14 |
|  | 3.37 | 1.21 | 0.13 | 43.85 | 6.51 | 2.55 | 1.85 | 10.91 | 65.66 | 29.05 | 25.55 | 3.50 | 0.22 | 4.60 | 0.05 | 1.33 | 0.21 | 0.28 | 101.07 |
|  | 3.38 | 1.52 | 0.07 | 43.97 | 6.51 | 2.50 | 1.77 | 10.78 | 65.53 | 28.77 | 25.34 | 3.43 | 0.17 | 4.60 | 0.04 | 1.45 | 0.16 | 0.31 | 100.98 |
| 7 | 7.05 | 3.10 | 0.08 | 44.73 | 5.95 | 0 | NR | 17.80 | 68.49 | 28.48 | 26.64 | 1.83 | NR | NR | NR | 2.16 | NR | 0.10 | N/A |
|  | 7.19 | 5.20 | 0.11 | 44.91 | 6.09 | 0 | NR | 18.95 | 69.95 | 28.95 | 27.50 | 1.45 | NR | NR | NR | 2.05 | NR | 0.09 | N/A |
|  | 6.17 | 4.25 | 0.08 | 41.44 | 5.76 | 0 | NR | 14.56 | N/A | 21.88 | 20.37 | 1.51 | NR | NR | NR | 3.16 | NR | 0.13 | N/A |
| 8 | 5.26 | 2.05 | 0.05 | 45.44 | 5.84 | 0.47 | 1.45 | 6.15 | 59.35 | 27.00 | 25.83 | 1.17 | 1.34 | 6.25 | 0.21 | 4.32 | 0.25 | 0.27 | 99.57 |
|  | 5.27 | 1.96 | 0.04 | 45.82 | 5.85 | 0.51 | 1.68 | 6.63 | 60.49 | 27.08 | 25.93 | 1.15 | 1.08 | 6.25 | 0.17 | 4.52 | 0.20 | 0.37 | 100.60 |
|  | NR | NR | NR | 45.63 | 5.67 | 0.53 | 1.73 | 6.63 | 60.18 | 26.67 | 25.54 | 1.14 | 1.46 | 6.25 | 0.23 | 4.47 | 0.21 | 0.23 | N/A |
| 9 | 3.95 | 0.52 | 0.05 | 43.41 | 5.08 | 0.36 | 1.50 | 10.48 | 60.83 | 27.86 | 27.24 | 0.62 | NR | NR | NR | 2.02 | 0.07 | 0.13 | 95.25 ^a^ |
|  | 4.69 | 0.88 | 0.06 | 43.65 | 5.08 | 0.36 | 1.72 | 10.51 | 61.33 | 29.06 | 28.47 | 0.59 | NR | NR | NR | 1.81 | 0.07 | 0.05 | 97.84 ^a^ |
|  | NR | NR | NR | 43.50 | 5.04 | 0.29 | 1.69 | 9.85 | 60.37 | 27.67 | 26.92 | 0.75 | NR | NR | NR | 1.55 | 0.04 | 0.07 | N/A |
| 10 | 4.18 | 1.72 | 0.02 | 43.03 | 5.99 | 0.63 | 2.21 | 8.02 | 59.88 | 31.92 | 27.66 | 4.26 | NR | NR | NR | 0.90 | 0.16 | 0.32 | 98.76 ^a^ |
|  | 3.63 | 1.70 | 0.01 | 42.22 | 5.94 | 0.63 | 2.14 | 8.45 | 59.38 | 32.45 | 28.09 | 4.35 | NR | NR | NR | 0.97 | 0.16 | 0.34 | 98.29 ^a^ |
|  | NR | NR | NR | 42.06 | 5.90 | 0.44 | 1.26 | 5.97 | 55.64 | 30.86 | 27.34 | 3.53 | NR | NR | NR | 0.97 | 0.16 | 0.33 | N/A |
| 11 | 3.46 | 1.56 | 0.44 | 43.14 | 6.02 | 2.29 | 4.18 | 10.26 | 65.88 | 28.39 | 24.81 | 3.58 | 0.23 | 4.60 | 0.05 | 1.35 | -0.17 | 0.06 | 100.69 |
|  | 3.16 | 1.49 | 0.37 | 43.09 | 6.03 | 2.30 | 4.83 | 9.02 | 65.27 | 28.50 | 24.83 | 3.66 | 0.18 | 4.60 | 0.04 | 1.35 | -0.19 | 0.04 | 99.76 |
|  | 3.17 | 1.49 | 0.54 | 43.10 | 6.02 | 2.28 | 4.26 | 10.09 | 65.76 | 28.62 | 24.94 | 3.68 | 0.18 | 4.60 | 0.04 | 1.34 | -0.27 | 0.02 | 100.29 |
| 12 | 2.68 | 1.89 | 0 | 44.78 | 4.84 | 1.07 | 1.72 | 7.83 | 60.23 | 29.31 | 28.97 | 0.34 | 1.04 | 5.80 | 0.18 | NR | 0.24 | 0.22 | N/A |
|  | 3.08 | 1.96 | 0 | 44.93 | 4.84 | 1.11 | 1.71 | 7.57 | 60.18 | 24.81 | 24.49 | 0.33 | 0.93 | 5.80 | 0.16 | NR | 0.25 | 0.23 | N/A |
|  | 2.87 | 2.06 | 0 | 44.70 | 4.96 | 1.10 | 1.70 | 7.84 | 60.30 | 27.09 | 26.74 | 0.35 | 0.87 | 5.80 | 0.15 | NR | 0.25 | 0.24 | N/A |
| 13 | 4.00 | 1.30 | 0 | 40.30 | 5.80 | 1.1 | 2.10 | 9.80 | 59.10 | 27.00 | 24.10 | 2.90 | 0.40 | 6.25 | 0.06 | 1.40 | 0.40 | 0.20 | 93.60 |
|  | 5.40 | 1.30 | 0 | 39.50 | 5.80 | 1.2 | 2.00 | 9.50 | 58.00 | 27.10 | 24.20 | 3.00 | 0.50 | 6.25 | 0.08 | 1.40 | 0.40 | 0.10 | 94.10 |
|  | 4.40 | 1.40 | 0 | 39.20 | 5.70 | 1.3 | 2.00 | 9.40 | 57.60 | 27.00 | 24.00 | 3.00 | 0.50 | 6.25 | 0.08 | 1.40 | 0.40 | 0.30 | 92.70 |
| 14 | 3.29 | 1.44 | 0.49 | 43.67 | 6.85 | 1.78 | 3.08 | 10.76 | 66.14 | 28.35 | NR | NR | 0.18 | 4.60 | 0.04 | 1.50 | 0.00 | 0.11 | 100.90 |
|  | 3.42 | 1.59 | 0.84 | 44.35 | 6.99 | 1.82 | 3.18 | 10.91 | 67.25 | 28.52 | NR | NR | 0.18 | 4.60 | 0.04 | 1.52 | 0.00 | 0.11 | 102.48 |
|  | 3.37 | 1.49 | 0.65 | 43.46 | 6.87 | 1.80 | 3.08 | 10.71 | 65.92 | 27.75 | NR | NR | 0.23 | 4.60 | 0.05 | 1.51 | 0.00 | 0.17 | 100.27 |
| mean | 3.89 | 1.64 | 0.13 | 43.68 | 6.12 | 1.05 | 2.14 | 10.44 | 63.31 | 28.10 | 26.18 | 1.91 | 0.70 | N/A | 0.12 | 1.74 | 0.15 | 0.23 | 99.39 |
| stdev | 1.23 | 0.94 | 0.22 | 1.88 | 0.85 | 0.82 | 1.08 | 2.75 | 3.75 | 2.04 | 2.06 | 1.49 | 0.44 | N/A | 0.07 | 1.00 | 0.16 | 0.12 | 2.61 |
| %RSD | 32 | 58 | 171 | 4.3 | 14 | 78 | 51 | 26 | 5.9 | 7.3 | 7.9 | 78 | 62 | N/A | 56 | 58 | 110 | 52 | 2.6 |

NR – not run

N/A – not applicable

^a^ Total value calculated without protein

^b^ Total value calculated using whole ash instead of extractives free ash
