# Supplementary material for: Compositional Analysis of Biomass Reference Materials: Results from an Interlaboratory Study
Source: Bioenergy Res. Author manuscript; Available in PMC 2016 Mar 25. (PMC4807399; doi:10.1007/s12155-015-9675-1)
Supplement: Supp4 [file NIHMS740646-supplement-Supp4.doc]

**Table S4** RM 8494 Wheat Straw. Individual lab compositional analysis results reported as mass fractions and are all reported on a 105°C dry-mass basis. Mean, standard deviation, and percent relative standard deviation (%RSD) are reported for the data set. Note that these summary statistics are calculated using each individual data point rather than the mean of the laboratory means

| Lab | Water Extr. | Ethanol Extr. | Sucrose | Glucan | Xylan | Arabinan | Galactan | Mannan | Struct. Sugars | Total Lignin | Acid-Insoluble Residue | Acid-Soluble Lignin | Protein | %N to Protein | %N | Acetyl Groups | Extr. Free Ash | Whole Ash | Total |
| --- | --- | --- | --- | --- | --- | --- | --- | --- | --- | --- | --- | --- | --- | --- | --- | --- | --- | --- | --- |
| 1 | 13.56 | 1.78 | 0.43 | 33.54 | 23.80 | 3.41 | 1.03 | 0.30 | 62.08 | 16.49 | 15.84 | 0.65 | NR | NR | NR | 1.02 | 5.75 | 9.93 | 100.69 ^a^ |
|  | 13.68 | 2.02 | 0.48 | 33.56 | 23.44 | 3.63 | 0.85 | 0.43 | 61.91 | 16.44 | 15.76 | 0.68 | NR | NR | NR | 1.14 | 5.68 | 9.92 | 100.87 ^a^ |
|  | 13.61 | 1.87 | 0.44 | 33.74 | 24.05 | 3.31 | 1.25 | 0.41 | 62.76 | 16.26 | 15.54 | 0.72 | NR | NR | NR | 0.97 | 5.71 | 9.92 | 101.19 ^a^ |
| 2 | NR | NR | NR | NR | NR | NR | NR | NR | N/A | NR | NR | NR | 5.1 | 6.25 | 0.82 | NR | NR | 10.5 | N/A |
|  | NR | NR | NR | NR | NR | NR | NR | NR | N/A | NR | NR | NR | 5.1 | 6.25 | 0.82 | NR | NR | 10.4 | N/A |
|  | NR | NR | NR | NR | NR | NR | NR | NR | N/A | NR | NR | NR | 5.0 | 6.25 | 0.80 | NR | NR | 10.7 | N/A |
| 3 | NR | NR | NR | 33.29 | 18.31 | 2.21 | 0.70 | 0.35 | 54.86 | 17.00 | 16.36 | 0.64 | NR | NR | NR | NR | NR | NR | N/A |
|  | NR | NR | NR | 33.63 | 19.46 | 2.20 | 0.66 | 0.81 | 56.76 | 16.95 | 16.33 | 0.62 | NR | NR | NR | NR | NR | NR | N/A |
|  | NR | NR | NR | 33.70 | 19.84 | 2.29 | 0.70 | 1.18 | 57.71 | 16.94 | 16.31 | 0.63 | NR | NR | NR | NR | NR | NR | N/A |
| 4 | 9.92 | 1.62 | 0.21 | 34.74 | 18.62 | 0.26 | 1.11 | 0 | 54.72 | 18.33 | 14.12 | 4.21 | 1.95 | 4.60 | 0.42 | 1.55 | 6.48 | 10.13 | 94.57 |
|  | 9.93 | 1.49 | 0.32 | 38.67 | 19.58 | 1.33 | 0.19 | 0 | 59.76 | 18.70 | 14.47 | 4.23 | 1.86 | 4.60 | 0.40 | 1.76 | 6.57 | 10.04 | 100.07 |
|  | 9.90 | 1.57 | 0.15 | 36.47 | 18.89 | 1.47 | 0.11 | 0 | 56.94 | 18.33 | 14.05 | 4.28 | 2.15 | 4.60 | 0.47 | 0.96 | 6.60 | 10.16 | 96.45 |
| 5 | 14.09 | 1.41 | 0.76 | 35.35 | 22.05 | 2.50 | 1.74 | 0.57 | 62.21 | 14.29 | 13.44 | 0.85 | 4.29 | 6.25 | 0.69 | 3.73 | NR | 10.46 | N/A |
|  | 14.21 | 1.24 | 0.83 | 35.44 | 22.38 | 3.15 | 1.72 | 0.12 | 62.81 | 14.19 | 13.34 | 0.85 | 4.40 | 6.25 | 0.70 | 3.51 | NR | 10.70 | N/A |
|  | outlier | 1.42 | 0.93 | 34.13 | 21.18 | 2.40 | 1.67 | 0.71 | 60.08 | 14.13 | 13.24 | 0.89 | 4.50 | 6.25 | 0.72 | 3.58 | NR | 10.60 | N/A |
| 6 | 16.17 | 1.71 | 1.82 | 33.05 | 18.95 | 1.23 | 2.45 | 0 | 55.67 | 18.11 | 14.14 | 3.97 | 2.48 | 4.60 | 0.54 | 2.10 | 6.06 | 9.76 | 102.30 |
|  | 16.02 | 2.06 | 1.64 | 32.66 | 19.08 | 1.32 | 2.45 | 0 | 55.51 | 18.06 | 13.92 | 4.14 | 2.53 | 4.60 | 0.55 | 2.06 | 5.77 | 10.01 | 102.01 |
|  | 15.77 | 2.13 | 1.54 | 32.81 | 19.38 | 1.33 | 2.48 | 0 | 55.99 | 18.25 | 14.13 | 4.12 | 2.48 | 4.60 | 0.54 | 1.96 | 5.85 | 9.95 | 102.44 |
| 7 | 16.54 | outlier | 6.82 | 41.71 | 22.55 | 3.55 | NR | 1.01 | 68.83 | 22.95 | 18.46 | 4.50 | NR | NR | NR | 1.83 | NR | outlier | N/A |
|  | 19.89 | 13.85 | 7.31 | 36.05 | 19.27 | 3.32 | NR | 2.50 | 61.14 | 22.94 | 18.60 | 4.34 | NR | NR | NR | 1.94 | NR | 8.55 | N/A |
|  | 16.97 | 15.24 | 7.93 | outlier | outlier | 2.20 | NR | 1.64 | N/A | 22.37 | 18.95 | 3.42 | NR | NR | NR | outlier | NR | 8.12 | N/A |
| 8 | 19.51 | 4.27 | 1.16 | 35.18 | 19.24 | 2.08 | 0.32 | 0 | 56.82 | 22.00 | 19.35 | 2.65 | 1.74 | 6.25 | 0.28 | 5.38 | 7.18 | 9.36 | 116.90 |
|  | 18.99 | 4.56 | 1.02 | 34.55 | 19.54 | 2.09 | 0.29 | 0 | 56.47 | 22.72 | 20.14 | 2.58 | 2.30 | 6.25 | 0.37 | 5.52 | 6.68 | 9.34 | 117.23 |
|  | NR | NR | NR | 29.95 | 17.65 | 1.88 | 0.26 | 0 | 49.75 | 23.09 | 20.38 | 2.71 | 2.41 | 6.25 | 0.39 | 5.44 | 6.48 | 9.22 | N/A |
| 9 | 12.71 | 2.67 | 1.23 | 34.46 | 19.83 | 2.71 | 0.26 | 0 | 57.26 | 20.09 | 17.53 | 2.56 | NR | NR | NR | 3.21 | 6.76 | 10.19 | 102.70 ^a^ |
|  | 13.17 | 4.50 | 1.36 | 34.21 | 19.49 | 2.75 | 0.25 | 0 | 56.69 | 21.71 | 18.78 | 2.93 | NR | NR | NR | 3.44 | 7.15 | 9.94 | 106.65 ^a^ |
|  | NR | NR | NR | 33.57 | 18.44 | 2.16 | 0.25 | 0 | 54.42 | 20.19 | 17.49 | 2.70 | NR | NR | NR | 3.22 | 7.00 | 10.24 | N/A |
| 10 | 12.44 | 2.00 | 0.91 | 32.83 | 20.29 | 3.20 | 0 | 0 | 56.32 | 19.96 | 16.46 | 3.49 | NR | NR | NR | 2.38 | 6.70 | 11.02 | 99.80 ^a^ |
|  | 12.25 | 3.28 | 0.99 | 33.15 | 20.84 | 2.93 | 0 | 0 | 56.93 | 20.10 | 16.56 | 3.54 | NR | NR | NR | 2.43 | 6.49 | 11.10 | 101.48 ^a^ |
|  | NR | NR | NR | 32.31 | 19.83 | 2.85 | 0 | 0 | 54.99 | 20.32 | 16.52 | 3.80 | NR | NR | NR | 2.41 | 6.91 | 10.81 | N/A |
| 11 | 15.94 | 1.99 | 2.00 | 33.13 | 19.58 | 1.01 | 2.33 | 0 | 56.04 | 17.76 | 13.73 | 4.04 | 1.36 | 4.60 | 0.30 | 2.01 | 4.98 | 9.68 | 100.09 |
|  | 16.33 | 1.99 | 1.96 | 33.33 | 19.55 | 0.98 | 2.29 | 0 | 56.15 | 17.64 | 13.62 | 4.02 | 1.39 | 4.60 | 0.30 | 1.98 | 5.25 | 9.57 | 100.74 |
|  | 16.13 | 1.93 | 2.05 | 33.76 | 19.77 | 1.00 | 2.31 | 0 | 56.83 | 18.14 | 14.12 | 4.02 | 1.36 | 4.60 | 0.29 | 2.02 | 5.19 | 9.45 | 101.60 |
| 12 | 10.18 | 6.07 | 1.34 | 34.28 | 17.80 | 1.56 | 0.55 | 0.40 | 54.58 | 15.10 | 13.48 | 1.62 | 3.54 | 5.80 | 0.61 | NR | 7.33 | 6.15 | N/A |
|  | 11.54 | 4.41 | 1.56 | 34.19 | 17.72 | 1.84 | 0.55 | 0.41 | 54.70 | 15.65 | 14.02 | 1.63 | 3.60 | 5.80 | 0.62 | NR | 7.37 | 7.03 | N/A |
|  | 9.90 | 6.29 | 1.30 | 34.39 | 17.61 | 1.94 | 0.54 | 0.41 | 54.88 | 16.35 | 14.76 | 1.58 | 4.52 | 5.80 | 0.78 | NR | 6.56 | 6.25 | N/A |
| 13 | 18.90 | 1.30 | 2.20 | 30.30 | 17.20 | 1.70 | 0.90 | 0.20 | 50.30 | 13.40 | 12.60 | 0.80 | 1.90 | 6.25 | 0.30 | 1.90 | 3.80 | 9.50 | 91.50 |
|  | 19.30 | 1.30 | 2.30 | 30.60 | 17.30 | 1.80 | 0.90 | 0.20 | 50.80 | 14.70 | 13.60 | 1.10 | 2.10 | 6.25 | 0.34 | 2.00 | 4.90 | 9.70 | 95.10 |
|  | 20.80 | 1.40 | 2.30 | 29.80 | 17.10 | 1.70 | 0.90 | 0.20 | 49.70 | 13.50 | 12.80 | 0.80 | 2.00 | 6.25 | 0.32 | 2.00 | 3.90 | 9.50 | 93.30 |
| 14 | 16.43 | 2.03 | 7.69 | 39.38 | 21.86 | 2.54 | 0.00 | 0.00 | 63.78 | 18.43 | NR | NR | 2.58 | 4.60 | 0.56 | 2.51 | 6.34 | 9.53 | 112.10 |
|  | 16.53 | 2.04 | 7.44 | 39.01 | 21.60 | 2.52 | 0.00 | 0.00 | 63.13 | 18.68 | NR | NR | 2.58 | 4.60 | 0.56 | 2.51 | 6.59 | 9.73 | 112.06 |
|  | 16.52 | 2.06 | 7.61 | 38.41 | 21.51 | 2.55 | 0.00 | 0.00 | 62.47 | 18.34 | NR | NR | 2.62 | 4.60 | 0.57 | 2.37 | 6.20 | 9.56 | 110.58 |
| mean | 14.93 | 3.23 | 2.36 | 34.30 | 19.86 | 2.18 | 0.89 | 0.30 | 57.44 | 18.17 | 15.64 | 2.51 | 2.88 | N/A | 0.52 | 2.53 | 6.14 | 9.65 | 102.60 |
| stdev | 3.19 | 3.27 | 2.52 | 2.54 | 1.85 | 0.81 | 0.83 | 0.52 | 4.22 | 2.78 | 2.23 | 1.46 | 1.23 | N/A | 0.18 | 1.20 | 0.92 | 1.13 | 6.86 |
| %RSD | 21 | 101 | 107 | 7.4 | 9.3 | 37 | 94 | 173 | 7.3 | 15 | 14 | 58 | 43 | N/A | 35 | 48 | 15 | 12 | 6.7 |

NR – not run

N/A – not applicable

outlier – not reported, identified by laboratory as an unusual value

^a^ Total value calculated without protein

^b^ Total value calculated using whole ash instead of extractives free ash
